# Exposure of Lactating Dairy Cows to Acute Pre-Ovulatory Heat Stress Affects Granulosa Cell-Specific Gene Expression Profiles in Dominant Follicles

**DOI:** 10.1371/journal.pone.0160600

**Published:** 2016-08-17

**Authors:** Jens Vanselow, Andreas Vernunft, Dirk Koczan, Marion Spitschak, Björn Kuhla

**Affiliations:** 1 Institute of Reproductive Biology, Leibniz Institute for Farm Animal Biology (FBN), 18196 Dummerstorf, Germany; 2 Institute of Nutritional Physiology, Leibniz Institute for Farm Animal Biology (FBN), 18196 Dummerstorf, Germany; 3 Institute of Genome Biology, Leibniz Institute for Farm Animal Biology (FBN), 18196 Dummerstorf, Germany; 4 Institute for Immunology, University of Rostock, 18055 Rostock, Germany; Qingdao Agricultural University, CHINA

## Abstract

High environmental temperatures induce detrimental effects on various reproductive processes in cattle. According to the predicted global warming the number of days with unfavorable ambient temperatures will further increase. The objective of this study was to investigate effects of acute heat stress during the late pre-ovulatory phase on morphological, physiological and molecular parameters of dominant follicles in cycling cows during lactation. Eight German Holstein cows in established lactation were exposed to heat stress (28°C) or thermoneutral conditions (15°C) with pair-feeding for four days. After hormonal heat induction growth of the respective dominant follicles was monitored by ultrasonography for two days, then an ovulatory GnRH dose was given and follicular steroid hormones and granulosa cell-specific gene expression profiles were determined 23 hrs thereafter. The data showed that the pre-ovulatory growth of dominant follicles and the estradiol, but not the progesterone concentrations tended to be slightly affected. mRNA microarray and hierarchical cluster analysis revealed distinct expression profiles in granulosa cells derived from heat stressed compared to pair-fed animals. Among the 255 affected genes heatstress-, stress- or apoptosis associated genes were not present. But instead, we found up-regulation of genes essentially involved in G-protein coupled signaling pathways, extracellular matrix composition, and several members of the solute carrier family as well as up-regulation of *FST* encoding follistatin. In summary, the data of the present study show that acute pre-ovulatory heat stress can specifically alter gene expression profiles in granulosa cells, however without inducing stress related genes and pathways and suggestively can impair follicular growth due to affecting the activin-inhibin-follistatin system.

## Introduction

It has been reported in several studies that fertility in lactating dairy cows is depressed during the summer months in warm areas of the world (for review see [1]. However, due to global warming this will be also an increasing problem in temperate zones. In particular, heat stress (HS) conditions before and after breeding, and on the day of breeding, has been found to be associated with low non return rates [2]. Some studies suggest that the use of gonadotropins to induce follicular development and ovulation can decrease the severity of seasonal postpartum infertility in dairy cows [3]. Generally, heat stress during summer aggravates the negative energy balance (NEB) of early lactating cows, a frequently observed phenomenon in high yielding dairy cattle. Heat stress generally deteriorates the body condition score, specifically affects reproductive parameters such as follicle differentiation and alters the composition of the follicular fluid in particular the concentrations of fatty acids, thus suggestively leading to inferior oocyte quality and compromised granulosa cell functions [4]. Recently, it was shown in a bovine cell culture model that increased concentrations of oleic acid specifically affect morphological and physiological features and gene expression levels of granulosa cells thus altering their functionality [5]. In a retrospective study evaluating the effects of heat stress that was defined as temperatures ≥29°C on the conception rates after artificial insemination, it has been reported in lactating Holstein cows that the conception rate in fact was negatively affected by HS exposition prior, but not after artificial insemination, thus suggesting that in particular, HS prior to and immediately after artificial insemination should be avoided [6]. Interestingly, the proportion of pregnancy losses was not affected thus suggesting that HS is especially deleterious during late folliculogenesis.

Not surprisingly, also the efficiency of reproductive bio-techniques is negatively affected under heat stress conditions. In a previous study it has been shown that the developmental competence of oocytes collected at different seasons was significantly different with lowest blastocyst rates during summer [7]. According to this study, season-induced impairment of the oocyte developmental competence might be partly explained by altered oocyte mitochondrial functions. Studies with other species clearly point out that in particular the oocyte differentiation is highly sensitive to HS conditions. In sows it was demonstrated that oocytes had reduced competence and thus lower fertility during hot seasons [8]. In a mouse study it has been found that a subpopulation of the ovarian pool is highly sensitive to short term (1.5–2 h) maternal hyperthermia, but also to ex vivo HS conditions. In particular, the developmental competence of germinal vesicle (GV)-stage oocytes was disrupted in these experiments [9].

In a recent study it has been shown that pre- and post-partum cows respond to exposure to high ambient temperatures (28°C) kept under well-controlled climate chamber conditions with a significant decrease in feed intake and reduced daily and resting metabolic heat production compared to control animals housed under thermoneutral (15°C) conditions [10]. To distinguish the effect of heat from the unavoidable accompanying effects of reduced feed intake during higher ambient temperatures, a revised HS animal model with pair-fed (PF) cows as control group has been developed ensuring equal energy intake at different temperature conditions [11,12]. Here it could be shown that fat mobilization and fat oxidation of lactating cows were not affected despite heat-induced reduction of feed intake. But interestingly, cows under high ambient temperatures exhibited a metabolic shift towards a pronounced carbohydrate oxidation and extensive tissue protein degradation.

During the present study, a similar model comparing HS vs. PF control animals was used as previously described in [12] to analyze effects of acute pre-ovulatory HS on late folliculogenesis. As a first approach growth as well as the progesterone (P4) and estradiol (E2) contents of late pre-ovulatory dominant follicles, and the respective granulosa cell-specific gene expression profiles were analyzed using a genome-wide transcriptome analysis approach and bioinformatic evaluation.

## Materials and Methods

### Animals

**Fig 1:**
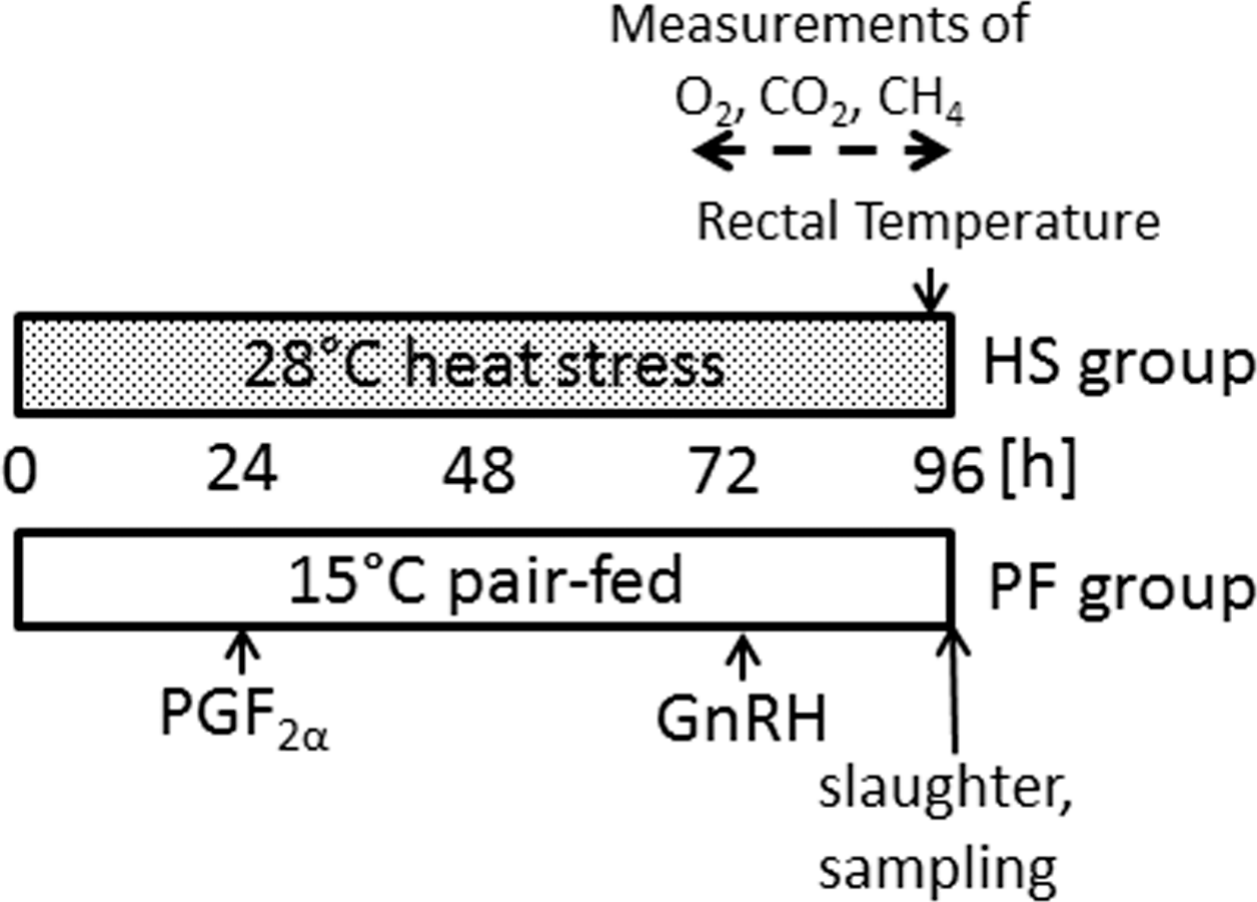
Experimental design of the present heat stress model. Non-pregnant German Holstein cows in established 2^nd^ lactation were randomly allocated to two groups: a PF control (n = 4) with a proportional feed restriction like it was measured in the HS group before, and an experimental HS group (n = 4). During the experimental period of 96h in a climate/respiration chamber at 28°C or 15°C the cows were synchronized with PGF_2α_ (Prostaglandin F2α) and subsequent GnRH injections prior to slaughter and sampling. Concentrations of O_2_, CO_2_ and CH_4_ in the chamber were measured during the last 24hrs before slaughter.

Eight non-pregnant German Holstein cows in established 2^nd^ lactation (245d±102d post-partum) and with estrous cycle activity as determined by ultrasonography using a transrectal 5 MHz linear transducer (L52 transducer, SonoSite Inc., USA) and a MicroMaxx ultrasound system (SonoSite Inc., USA) were identified from the herd at the Leibniz Institute for Farm Animal Biology (FBN). Cows were randomly allocated to two groups: a PF control (n = 4) with a proportional feed restriction like it was measured in the HS group before, and an experimental HS group (n = 4, Fig 1). Cows received a lactation diet based on corn and grass silage comparable to that described previously [12]. During the experiment animals were either kept in a respiration and climate chamber under thermoneutral (15°C, 64% relative humidiy, temperature humidity index (THI) = 60) or heat stress conditions (28°C, 52% relative humidiy, THI = 76) for a total of 95 hours (h). Concentrations of O_2_, CO_2_ and CH_4_ in the chamber were measured every 6 min during 24h before slaughter as described previously [12]. Daily metabolic heat production (HP) per metabolic body weight (mBW) was calculated from:

$$HP\;/\;mBW\;\left(kJ/kg^{0.75}\right)=16.18\;V_{O2}\left(L\right)+5.02\;V_{CO2}\left(L\right)-\;2.17\;V_{CH4}\left(L\right)+5.99\;Nu\;\left(g\right)\;/\;mBW\;kg^{0.75})$$

Urine N excretion (Nu) was not determined but assumed to be constant (50 g/d), thus accepting an error of about 10%. In the chamber, water and feed intake and milk yield were determined. For heat synchronization and to collect follicles of each animal at the late post-LH (luteinizing hormone), but still pre-ovulatory stage, cows were treated with 500 μg Prostaglandin F2α (PGF_2α_; PGF forte, Veyx Pharma GmbH, Schwarzenborn, Germany) to induce luteolysis of the functional corpus luteum 72 hrs before slaughter. Forty-nine hours later, the animals were injected with 100 μg of a GnRH (gonadotropin releasing hormone) analog (Depherelin, Gonavet Veyx, Veyx Pharma GmbH) to induce a preovulatory LH surge. At the time of PGF_2α_ and GnRH injections the sizes of follicles were estimated by ultrasonography. 22 hrs after GnRH injection rectal temperatures were measured and animals were transferred from the respiration chamber directly into the institutional slaughterhouse and slaughtered (23h after GnRH).

### Follicular fluid and granulosa cell collection

Immediately after slaughter ovaries were collected and the largest growing follicles of each animal were broadly dissected free from remaining ovarian tissue. Follicular fluid and basement membrane associated (i.e. mural) granulosa cells (mGC) were collected as described previously [13,14,15]. The follicular fluid together with cumulus-oocyte-complexes and free floating and only slightly adherent granulosa cells was harvested with a 18G needle by aspiration. The fluid was centrifuged (2 min, 400 rcf) and the cell-free supernatant was frozen and stored at −20°C for determination of steroid concentrations. mGC were isolated by cutting the follicle open with scissors and peeling off the follicular wall. The wall was then submersed in Ca^2+^ and Mg^2+^-free phosphate buffered saline (PBS, pH 7.4), and cells of the GC layer were gently scraped off with a small scalpel blade. Detached cell clots were collected with a pipette, the cells were isolated by centrifugation from the buffer, and the sedimented cells were frozen in liquid nitrogen and stored at −80°C until RNA preparation.

All experimental procedures were in accordance with the German Animal Welfare Act (TierSchG) in its respective edition and were approved by the local Animal Research Committee (Landesamt für Landwirtschaft, Lebensmittelsicherheit und Fischerei (LALLF) of Mecklenburg-West Pommerania, Germany (LALLF M-V/TSD/7221.3–1.1-074/12)).

### E2 and P4 Determination

Steroid concentrations of the follicular fluid were determined as described previously [14]. Briefly, P4 was determined using a competitive single-antibody 3H-radioimmunoassay (RIA). Follicular fluid of each sample (10 μl each) was diluted with assay buffer (90 μl). Ten microliters of the diluted sample was used to directly analyze (without extraction) P4 and E2 concentrations. The tracer, [1,2,6,7-3H]progesterone, was purchased from GE Healthcare (Freiburg, Germany). The antibody, raised in rabbits, was further purified by affinity chromatography on protein A superose (GE Healthcare). The intra- and inter-assay coefficients of variation (CVs) were 7.4% and 9.8%, respectively. Measurement of radioactivity was performed by a β-counter with integrated RIA calculation (TriCarb2900; Perkin Elmer, Rodgau, Germany). The concentration of E2 was estimated with an ultra-sensitive 125I-RIA (DSL, Sinsheim, Germany). The standard curve was established between 0.0025 and 0.750 ng/ml. Radioactivity was measured with an automatic gamma counter with integrated RIA calculation (Wizard; Perkin Elmer). The detection limit of the method was found to be 0.003 ng/ml. The intra- and inter-assay CVs were 8.4% and 10.2%, respectively.

### RNA preparation, cDNA synthesis and quantitative RT-PCR

For RNA preparation from frozen GC pellets the RNeasy mini kit with an integrated DNase 1 digestion step and QIAshredder homogenizers were applied according to the manufacturer´s (Qiagen, Hilden, Germany) advice. RNA quality was assessed with a Bioanalyzer Instrument (Agilent Technologies, St. Clara, CA, USA) thus resulting in mean RNA integrity values (RIN) of 7.8 (range: 7.1–8.5) and 7.9 (range: 7.0–9.0) for the HS and PF samples, respectively.

cDNA synthesis was performed with MMLV reverse transcriptase (GeneOn, Ludwigshafen, Germany) using oligo-(dT) primers (2 ng/μl) and random hexamer primers (4 ng/μl, both Roche, Mannheim, Germany). The cDNA was cleaned using the High Pure Purification Kit (Roche) and diluted in 50 μl of the provided elution buffer.

The abundance of selected transcripts was then determined by quantitative real-time PCR (qPCR) with SensiFastTM SYBR No-ROX (Bioline, Luckenwalde, Germany) and gene-specific primers (S1 Table). For the following reaction 0.25 and 0.5 μl cDNA were amplified in a total volume of 12 μl and the values of both were averaged considering different dilutions. The reaction was quantified in a LightCycler® 96 instrument (Roche, Mannheim, Germany) with following cycle conditions: pre-incubation at 95°C for 5 min, 40 amplification cycles of denaturation at 95°C for 20 s, annealing at 60°C for 15 s, extension at 72°C for 15 s, and a single-point fluorescence acquisition for 10 s. Melting point analysis was done immediately afterwards to ensure amplification of correct products as well as checking the length of PCR products by agarose gel electrophoresis (3%, ethidium bromide stained). Cloned PCR products were co-amplified as external standards. Of these, dilutions were freshly prepared to obtain five different concentrations of standards (5 x 10−12–5 x 10^−16^ g DNA/reaction). This enabled the absolute quantification of transcripts relative to the applied RNA/cDNA in order to assess the suitability of different housekeeping genes for normalization. Accordingly, the abundance of transcripts encoding *RPS18* (Ribosomal protein S18), *RPLP0* (Ribosomal protein, large, P0), *B2M* (Beta-2-microglobulin), *GAPDH* (Glyceraldehyde-3-phosphate dehydrogenase), *HPRT*1 (Hypoxanthine phosphoribosyltransferase 1), *TBP* (TATA box binding protein) and *HMBS* (Hydroxymethylbilane synthase) were determined in HS and PF samples. As shown in S2 Table three of these genes (*B2M*, *GAPDH*, *HPRT1*) showed considerable differences (fold change |FC| > 1.5) between both experimental groups even though not reaching levels of significance. On the other hand, *RPLP0* and *TBP* showed the least differences between both experimental groups. Because TBP was also recommended as an appropriate housekeeping gene for normalization in cultured GC under different conditions [16], this housekeeping gene was used during the present study for normalization. Thus transcript abundance levels determined by qPCR were expressed as values relative to TBP throughout this paper.

### Microarray profiling

Microarray analysis was performed with Bovine Gene 1.0 ST Arrays (Affymetrix, St. Clara, CA, USA). RNA samples were amplified and labeled using the “GeneChip® Expression 3’Amplification One-Cycle Target Labeling and Control Reagents” (Affymetrix) according to the supplier’s instructions. Hybridization was done overnight in the GeneChipR Hybridization Oven (Affymetrix) and visualized using the Affymetrix GeneChip Scanner 3000. The original data were further processed using the Expression Console (Version 1.3.1.187, Affymetrix). Normalization, background reduction and gene-level summary was performed using the Robust Multichip Average (RMA) procedure with default settings. Array results have been uploaded to the GEO database (GSE81737). Further comparative analysis of the data was realized with the Transcriptome Analysis Console 3.0 (Version 3.0.0.466, Affymetrix) using the integrated Analysis of Variance (ANOVA) statistical tool. The false discovery rate (FDR) procedure was also implemented using the Benjamini-Hochberg model [17]. The levels of significance for differential expression between the HS vs. PF samples were set with │FC│ >1.5 and P<0.05 according to ANOVA. For the hierarchical clustering default settings of the Transcriptome Analysis Console 3.0 was used.

### Statistics and bioinformatics evaluation

For statistical comparisons of various physiological data (Table 1), follicle diameters and steroid concentrations (Table 2), and of qPCR values of the two groups (HS vs. PF) the unpaired parametric t-test of the SigmaPlot 11.0 Statistical Analysis package (Jandel Scientific, San Rafael, CA, USA) was performed. For bioinformatic evaluation of the microarray results the Ingenuity pathway analysis (IPA) software (Qiagen) was used.

**Table 1:** Animal data of heat-stressed (HS) and pair-fed (PF) cows determined in the respiration chamber.

	HS	PF	p-value
Feed intake per kg BW [kg/kg*100]	4.24 ± 0.44	4.44 ± 0.60	0.797
Dry matter intake per kg BW [kg/kg*100]	1.64 ± 0.22	1.79 ± 0.13	0.561
Water intake per kg BW [L/kg*100]	9.1 ± 0.7	7.0 ± 0.7	0.08
Milk yield [kg]	22.9 ± 1.8	22.5 ± 2.1	0.891
Rectal Temperature [°C]	40.2 ± 0.4	38.4 ± 0.2	0.008
HP/ mBW [kJ/ kg^0.75^]	986.1 ± 32.8	931.3 ± 44.0	0.356

Values are means ± SEM. P, p-values according to unpaired t-tests; BW, body weight.

**Table 2:** Size of dominant follicles as determined by ultrasonography at the times of treatment with PGF_2α_ and GnRH, and intra-follicular steroid contents.

	Size PGF_2α_ [mm]	Size GnRH [mm]	P4 [ng/ml]	E2 [ng/ml]	P4/E2
PF	17.6±1.1	20.0±0.0	118.1±48.6	61.2±20.5	2.3±0.6
HS	17.3±1.3	18.3±1.0	107.1±9.3	32.4±6.4	3.7±0.7
p	0.83	0.14	0.42	0.11	0.19

Values are means ± SEM; n = 4 samples from each group; HS, heat stress group; PF, pair-fed control group; P, p-values according to unpaired t-tests.

## Results

### Feed intake, morphological and physiological parameters

As per design, HS and PF cows ingested comparable amounts of feed at the 4^th^ day of challenge when they were kept in a respiration chamber. Milk yield and metabolic heat production were also not different between groups (Table 1). However, water intake tended to be greater and rectal temperature was significantly higher in cows exposed to heat. Ultrasonographic examinations revealed nearly identical sizes of dominant follicles at the time of PGF_2α_ injections (heat induction) and slightly, but not significantly, reduced diameters of dominant follicles at the time of GnRH application (ovulation induction; Table 2) following HS. Considering intra-follicular steroid hormone concentrations, P4 levels were very similar in both groups, whereas the data suggest decreased E2 production in HS samples, however without reaching levels of significance. The P4/E2 ratios were clearly above 1 in all follicles, which is indicative for a late pre-ovulatory, E2-inactive stage after the LH surge [13]. Another reliable marker for an early post LH stage prior to ovulation is the expression of *PTGS2* transcripts encoding COX-2 [18,19]. According to the microarray data this transcript was indeed highly expressed in follicles of both groups with average hybridization signals of 1323 and 1722 in the PF and HS groups, respectively. According to ANOVA these values were not different between both groups (P = 0.3).

### Expression profiling

**Fig 2:**
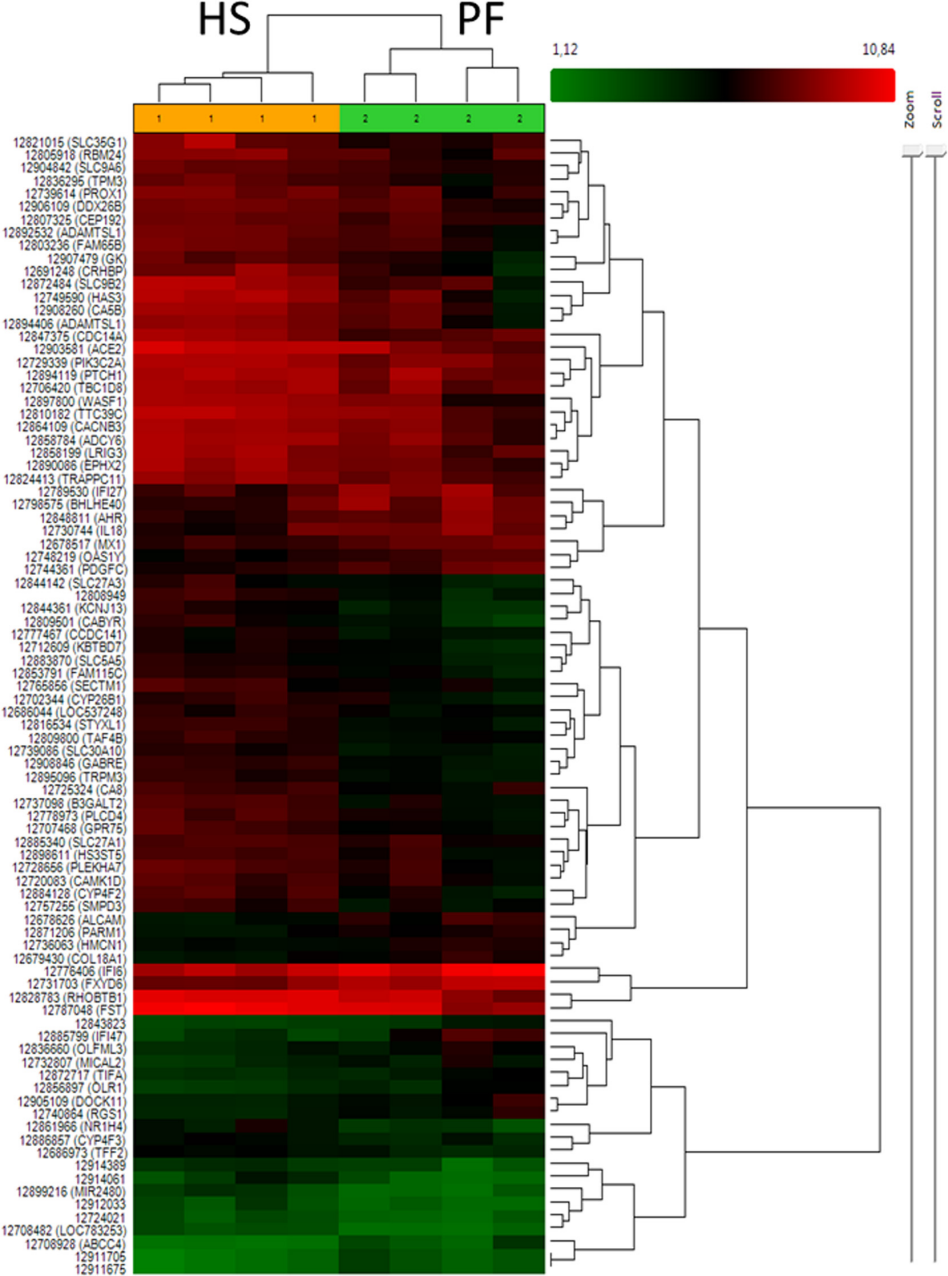
Hierarchical cluster analysis of heat affected genes. Based on the abundance of 88 differentially expressed granulosa cell transcripts (│FC│ >2 and unpaired One-Way ANOVA P<0.05) different samples were clustered using the respective tool of the Transcriptome Analysis Console software.

To identify transcripts that were affected by the increased ambient temperature, the levels of significance for differential expression between the HS vs. PF samples were set with │FC│>1.5 and P<0.05 according to unpaired One-Way ANOVA. Altogether, 304 different transcript clusters representing 255 annotated genes, among them 196 up-, but only 59 down-regulated, fulfilled these criteria (S3 Table). However, none of these transcript clusters reached the level of significance (p<0.05) according to FDR analysis. The fold change (FC) values of affected transcripts ranged from 5.4 to -7.8 (Table 3). Together with the relatively low number of affected transcripts this suggests that the effects of pre-ovulatory heat exposure did not induce large alterations of the GC gene expression profiles in dominant follicles. Nevertheless, hierarchical clustering revealed a clear separation of HS from PF samples according to the expression levels of the 88 mostly affected genes with │FC│ >2 and unpaired One-Way ANOVA P<0.05 (Fig 2). Considering the length of branches, which segregate individual samples this analysis showed a greater distance of PF samples from each other thus suggesting a greater variability between these samples compared to HS samples.

**Table 3:** qPCR re-evaluation of selected, differentially expressed transcripts according to microarray analysis.

Cluster ID	Gene Symbol	HS Signal	PF Signal	FC (array)	FC(qPCR)	P-valueqPCR	Description
12858784	*ADCY6*	519	239	2.2	3.5	0.03*	adenylate cyclase 6
12798575	*BHLHE40*	106	340	-3.2	-2.9	0.04*	basic helix-loop-helix family. member e40
12691248	*CRHBP*	251	77	3.5	6.4	0.02*	corticotropin releasing hormone binding protein
12787048	*FST*	1510	690	2.2	3.7	0.01*	follistatin
12907479	*GK*	204	73	2.7	1.1	0.67	glycerol kinase
12836105	*GNG12*	152	265	-1.7	-1.7	0.12	guanine nucleotide binding protein (G protein). gamma 12
12749590	*HAS3*	613	116	5.3	7.2	0.01*	hyaluronan synthase 3
12885799	*IFI47*	15	111	-7.8	-11.1	0.05*	interferon gamma inducible protein 47
12818707	*IFIT1*	15	26	-1.8	-1.5	0.10	interferon-induced protein with tetratricopeptide repeats 1
12690482	*MAP2K1*	198	104	1.9	1.8	0.05*	mitogen-activated protein kinase kinase 1
12678517	*MX1*	116	236	-2.0	-1.8	0.01*	myxovirus resistance 1
12748219	*OAS1Y*	77	159	-2.1	-2.3	0.04*	2'.5'-oligoadenylate synthetase 1. 40/46kDa
12744361	*PDGFC*	90	208	-2.2	-2.0	0.06	platelet derived growth factor C
12729339	*PIK3C2A*	630	290	2.2	3.0	0.02*	phosphatidylinositol-4-phosphate 3-kinase. catalytic 2 alpha
12778973	*PLCD4*	191	62	2.7	5.0	0.01*	phospholipase C. delta 4
12842793	*PRKACB*	626	350	1.9	1.7	0.25	protein kinase. cAMP-dependent. catalytic. beta
12793185	*PRKD1*	760	410	1.8	2.2	0.03*	protein kinase D1
12885340	*SLC27A1*	160	83	2.1	2.5	0.01*	solute carrier family 27 (fatty acid transporter). member 1

Mean hybridization values from microarray analysis and relative values (normalized to *TBP* expression) from qPCR analysis, fold changes (FC) of differences and p-values from unpaired t-tests are shown. Transcripts with significantly different expression levels according to qPCR re-evaluation are marked (*) and underlined.

Because of the relatively low levels of heat stress induced alterations and non-significant FDR p values, the abundance of 18 of the differentially expressed transcripts (according to ANOVA and FC thresholds) was re-evaluated by qPCR. Seven up- and 11 down-regulated transcripts were selected for qPCR among them the two top regulated genes (*HAS3* and *IFI47*), and a gene with established functional relevance during follicle development (*FST*), genes involved in the immune system (*IFIT1*, *OAS1Y*, *MX1*), in hormonal, intracellular and transcriptional signaling (*ADCY6*, *BHLHE40*, *CRHBP*, *GNG12*, *MAP2K1*, *PIK3C2A*, *PDGFC*, *PLCD4*, *PRKACB*, *PRKD1*), and in fat metabolism and transport (*GK*, *SLC27A1*). The tendency of regulation (i.e. either increased or decreased) was the same according to both, microarray and qPCR analysis. In addition, significantly different expression levels (according to unpaired t-tests) could be clearly confirmed for 13 out of the 18 transcripts by qPCR (Table 3).

Considering individual regulated transcripts *HAS3* encoding hyaluronan synthase 3 showed strongest up-regulation and *IFI47* (interferon gamma inducible protein 47) strongest down-regulation (Table 4). Also 10 members of the solute carrier family (*SLC11A2*, *SLC27A1*, *SLC27A3*, *SLC29A2*, *SLC30A10*, *SLC35E2*, *SLC35G1*, *SLC5A5*, *SLC9A6*, *SLC9B2*) showed significant up-regulation with FC values between 1.5 to 3.8 (S3 Table). Differential expression of *SLC27A1* was confirmed by qPCR (Table 3). Most interestingly, *FST* encoding follistatin showed clear up-regulation under heat stress conditions (S3 Table and Table 3). Follistatin is a member of the activin-inhibin-follistatin system, can directly bind to and thus neutralize activin and was originally described as a specific follicle-stimulating hormone (FSH) -release inhibitor [20]. Remarkably, except for *HSPA12A* (heat shock 70kDa protein 12A) no other known genes involved in heat stress reactions as heat shock factors or other heat shock proteins were affected although most of them are clearly present at different expression levels in the cells. Also genes involved in steroidogenesis (e.g. *CYP11A1*, *CYP19A1*, *HSD3B1*, *HSD17B10*, *HSD17B4*, and *HSD17B8*) were present at considerably high levels, but were virtually unaffected. Pro-apoptotic (*CASP1 to CASP9*, *BAX*, *BAD*, *BOK*) or anti-apoptotic transcripts (*BCLs*) were also not significantly affected. Except for *CCNG2* transcripts encoding cyclin G2 (S3 Table) no other regulators of the cell cycle or the proliferation marker *PCNA* (proliferating cell nuclear antigen) were found regulated.

**Table 4:** Top 20 regulated genes according to microarray analysis. Mean hybridization signals and fold change (FC) differences are shown.

Transcript Cluster ID	Gene Symbol	PF Signal	HS Signal	FC	Description
12749590	HAS3	116	617	5.4	hyaluronan synthase 3
12737098	B3GALT2	44	177	4.1	UDP-Gal:betaGlcNAc beta 1,3-galactosyltransferase
12884128	CYP4F2	49	190	3.9	cytochrome P450, family 4, subfamily F, polypeptide 2
12872484	SLC9B2	153	584	3.8	solute carrier family 9, subfamily B, member 2
12844361	KCNJ13	22	82	3.7	potassium inwardly-rectifying channel, subfamily J, member 13
12899216	MIR2480	4	15	3.7	microRNA mir-2480
12894406	ADAMTSL1	113	416	3.7	ADAMTS-like 1
12809501	CABYR	26	91	3.5	calcium binding tyrosine-(Y)-phosphorylation regulated
12691248	CRHBP	77	269	3.5	corticotropin releasing hormone binding protein
12908260	CA5B	153	512	3.4	carbonic anhydrase VB, mitochondrial
12776406	IFI6	1458	648	-2.2	interferon, alpha-inducible protein 6
12708928	ABCC4	8	3	-2.4	ATP-binding cassette, sub-family C (CFTR/MRP), member 4
12789530	IFI27	391	158	-2.5	putative ISG12(a) protein
12836660	OLFML3	57	22	-2.6	olfactomedin-like 3
12856897	OLR1	39	14	-2.8	oxidized low density lipoprotein (lectin-like) receptor 1
12678626	ALCAM	124	43	-2.9	activated leukocyte cell adhesion molecule
12730744	IL18	251	86	-2.9	interleukin 18 (interferon-gamma-inducing factor)
12731703	FXYD6	719	241	-3.0	FXYD domain containing ion transport regulator 6
12798575	BHLHE40	340	108	-3.2	basic helix-loop-helix family, member e40
12885799	IFI47	111	14	-7.8	interferon gamma inducible protein 47

### Bioinformatic evaluation

**Fig 3:**
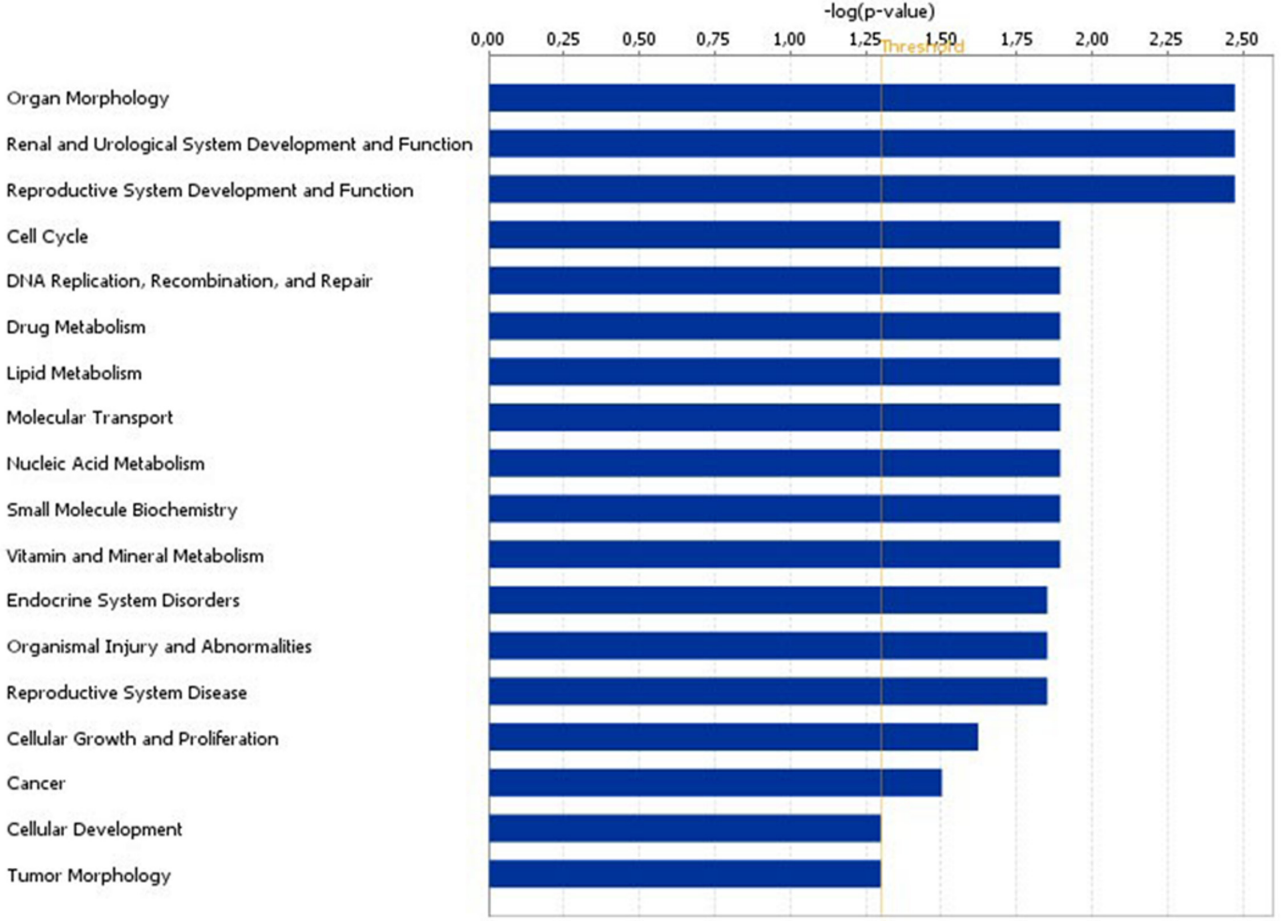
Affected “Diseases and Functions” in granulosa cells under acute pre-ovulatory heat stress conditions. Only significantly affected functions with p < 0.05 (indicated threshold line) according to bioinformatics IPA analysis were shown.

From the 304 differentially regulated transcript clusters 223 could be recognized and analyzed by IPA. These could be significantly assigned to 18 “Diseases and Functions”, of which “Reproductive System Development and Function”, “Cell Cycle”, “Endocrine System Disorders”, “Reproductive System Disease”, and “Cellular Growth and Proliferation” seem to be the most interesting in the functional context of folliculogenesis (Fig 3). Also 38 IPA Canonical Pathways were predicted as affected in GC by heat stress exposure of the animals (S4 Table). Most interestingly, transcripts encoding enzymes, which are essentially involved in G-protein coupled signaling, were found affected by heat stress. The heat affected transcripts *PLCD4* and *PIK3C2A* encoding the enzymes “phospholipase C, delta 4” and “phosphatidylinositol-4-phosphate 3-kinase, catalytic subunit type 2 alpha” were assigned to 14 and even 21 specific pathways, respectively. Both are essential factors of the phosphatidylinositol G-Protein signaling system, which is for example essentially involved in mediating oxytocin actions, but also in a plethora of other intracellular signaling processes. Another heat affected transcript was *ADCY6* encoding “adenylate cyclase 6”. This enzyme is also essentially involved in the cAMP/PKA (protein kinase A) mediated G-Protein pathway, which is essential for FSH and LH receptor signaling, and was found assigned to 12 specific pathways. Differential expression of these transcripts was confirmed by qPCR. Also *PDGFC* (platelet derived growth factor C), *PDGFB* (platelet-derived growth factor beta polypeptide), *SLC27A1* (solute carrier family 27 (fatty acid transporter), member 1) and *SLC27A3* (solute carrier family 27 (fatty acid transporter), member 3) were assigned to numerous different pathways (12, 12, 8 and 7, respectively).

## Discussion

### Acute pre-ovulatory heat stress tends to compromise growth of the dominant follicle

As intended by the present experimental design animals of the HS and PF control group did not show considerable differences of most physiological parameters except for body (rectal) temperature. Considering feed and dry matter intake, and even milk yield and heat production, we found no significant differences of means between experimental and control groups. Water intake tended to be higher under heat stress, which can certainly be explained by the need of heat stressed animals to control their body temperature. This suggests that the observed alterations of reproductive parameters can be purely assigned to heat stress without collateral effects due to reduced feed intake. The remarkably increased rectal temperature clearly indicates that the animals actually are not able to maintain temperature homoestasis under the present acute HS conditions. Nevertheless, we found only moderate and not significant effects of acute pre-ovulatory heat stress on the growth of dominant follicles. In contrast, in a former study in lactating Holstein cows it has been shown that dominant, but not subordinate follicles of animals in a non-shade management are smaller compared to those in a shade management system at day 8, but not at earlier days of the cycle [21]. From their data the authors conclude that HS may alter the efficiency of follicle selection and dominance. In another study in bos indicus, even long term exposure to HS (28 days, 30°C) had no immediate effects on reproductive functions, as on follicular recruitment or on the pattern of follicular growth in Phases I and II., but exerted delayed deleterious effects on ovarian follicular dynamics and oocyte competence [22]. This may indicate that this cattle breed shows a better ability to adapt to heat stress conditions. On the other hand, long term heat exposure cannot be directly compared with the present acute HS model, where we found only moderate and not-significant effects on late follicular growth at best. However, it is still interesting that GC derived *FST* transcripts encoding follistatin clearly showed significantly higher expression levels in animals of the HS group. This was confirmed by the genome-wide microarray data as well as by qPCR re-evaluation. Follistatin was originally discovered as a FSH inhibiting substance present in ovarian follicular fluid [20]. Now we know that it directly binds to activin [23] as a member of the activin-inhibin-follistatin system thus neutralizing activin´s actions [24]. In a previous long term heat stress experiment however, comparing heat stressed and cooled animals throughout the complete estrous cycle, increased plasma levels of FSH have been reported in cooled animals [25]. During the present study FSH levels have not been determined, but increased expression of follistatin clearly suggests reduced action of FSH on GC due to reduced release of FSH and also LH from the pituitary. Besides direct effects on FSH release from gonadotropic neurons (see above) it has been shown that follistatin may also have indirect effects on LH release via inhibiting activin´s actions in hypothalamic GnRH neurons [26]. According to the established timing of folliculogenesis and the experimental design, it can be expected that animals were exposed to heat stress during the follicular phases of dominance after selection, which is characterized by only moderate follicular growth. Accordingly, reduced levels of FSH, but in particular of LH during this late pre-ovulatory stage could exert relevant effects on the growth of dominant follicles. Besides these endocrine actions it is also well known that follistatin can act as a paracrine or autocrine antagonist of activin within the bovine follicle in addition. *FST* transcripts are highly up-regulated in large E2-active follicles [27]. Whereas activin facilitates FSH-induced granulosa cell proliferation and mitogenesis [28], it has been shown in a recent study that follistatin induces caspase3-dependent apoptosis through Bcl2/Bax gene family members in bovine GC [29], probably by antagonizing activin´s actions. Significant alterations of apoptosis related transcripts however, were not observed within the analyzed mural GC fraction, thus challenging the view that acute HS generally induces apoptosis among these cells via paracrine or autocrine actions of GC derived follistatin. Taken together, these data suggest that increase levels and endocrine actions of follistatin from the GC layer could reduce the FSH, but in particular the LH levels and thus indirectly compromise the late growth phase of dominant follicles. This in turn might then also lead to reduced efficiency of follicular selection as discussed in previous studies on heat stress effects [21,30,31]. Interestingly, it has been found recently, that the expression of the components of the activin–inhibin–follistatin system is also altered in bovine ovarian cysts [32], thus suggesting the initiation of similar unfavorable processes within the dominant follicle by acute pre-ovulatory heat stress.

HS effects on steroidogenesis, however, were less clear. The P4 production seemed completely unaffected, which is in line with the observation that the abundance of steroidogenic transcripts seemed virtually unaffected in corresponding cells of the granulosa. The intra-follicular E2 content tended to be lower, however without statistical significance. In previous studies contradictory data have been reported on follicular steroid production under heat stress conditions. In a similar animal model (i.e. 7 days HS in an environmental chamber) E2 and P4 production was found unchanged in dominant follicles [31], whereas in another study it was reported that acute heat stress exposure during the winter season reduced the E2 concentrations [33], which is also in line with the observation that E2 production was lower during the hot (autumn and summer) compared to the cool season (winter). However, a smaller diameter and lower steroid concentrations in pre-ovulatory follicles lead to a smaller corpus luteum and a lower progesterone plasma concentration in the later dioestrus and therefore may compromise implantation and development of an embryo [34]. But a larger number of animals however, might be necessary in future experiments to confirm the relatively weak effects of acute HS during the late follicular growth phase.

### Acute pre-ovulatory heat stress induces altered gene expression profiles in granulosa cells

Genome wide expression analysis by mRNA microarrays revealed that acute pre-ovulatory heat stress had only moderate, but specific effects on the granulosa cell transcriptome. Only a small subset of genes was affected with relatively moderate alterations (fold changes). Because none of the transcript clusters reached the levels of significance according to FDR analysis (p < 0.05), we re-evaluated 18 selected transcripts by qPCR and could confirm differential expression of about two thirds of these genes. Thus not all of the microarray could be clearly affirmed, most likely due to considerable variations between samples and generally low fold change levels. However, hierarchical cluster analysis unambiguously indicated distinct expression profiles in GC samples from HS vs. PF animals. This is clearly indicative of specific effects of acute pre-ovulatory HS on the granulosa cell transcriptome. Interestingly, the variation among the PF samples was even greater than among HS samples thus suggesting directed alterations resulting from heat exposure.

In detail we found highest up-regulation in case of *HAS3* transcripts encoding “hyaluronan synthase 3”. *HAS3* is one of three mammalian hyaluronan synthase genes, *HAS1*, *HAS2*, and *HAS3* [35], which are involved in the synthesis of the unbranched glycosaminoglycan hyaluronan (HA), a major constituent of the extracellular matrix. It has been shown that increased HA production by cancer cells promoted their invasive capacity [36]. In the ovary, HA produced by the granulosa layer are essentially involved in the formation of the follicular fluid and play a role during cumulus oocyte expansion [37,38]. In cow, but also in horse in particular the expression of *HAS2* is strongly induced by gonadotropins in GC of pre-ovulatory follicles [39,40]. In the rat *Has1* and *Has2* transcripts are expressed in cells of the theca and granulosa, whereas HA molecules are predominantly localized in the extracellular matrix of the theca layer, within the follicular fluid, but also the zona pellucida of large antral follicles [41]. Interestingly, also *B3GALT2* transcripts, encoding the membrane-bound glycoprotein enzyme “UDP-Gal:betaGlcNAc beta 1,3-galactosyltransferase”, which is involved in the modification of cell surface molecules and thus cell-cell interactions were strongly up-regulated in GC of the HS group. In a recent paper it has been shown that a similar enzyme, “core 1 β1, 3-galactosyltransferase 1” (C1galt1) synthesizing “core 1-derived O-glycans” is functionally involved in cumulus expansion [42].

The functional relevance of *HAS3* up-regulation after acute pre-ovulatory heat stress is not clear yet, however, one might speculate that the production of glycan molecules and thus the modulation of the cell-cell interactions within cells of the granulosa are significantly altered by HS exposure.

Another highly up-regulated gene was *CRHBP* encoding “corticotropin releasing hormone binding protein” (Table 3). This factor was found expressed in the primate corpus luteum, where it was up-regulated during the late luteal phase and might play a role for luteal development [43]. It was also found expressed in cells of the granulosa and theca. In humans *CRHBP* could be identified in most female reproductive tissues, where it is involved in follicular maturation, ovulation, and luteolysis [44]. CRHBP mRNA increased in the primate corpus luteum (CL) after LH withdrawal [45]. However, the functional relevance of this factor during late folliculogenesis and in particular its remarkable up-regulation under acute pre-ovulatory HS conditions in bovine GC is not clear yet.

Also ten members of the large solute carrier family were among the affected genes (S3 Table) with all of them up-regulated and one of them, *SLC9B2*, among the most up-regulated transcripts (Table 4). Several members of this protein family are expressed in the GC layer. Two of them, “solute carrier family 2 member 1” and “solute carrier family 2 member 3” have been suggested as cumulus cell derived molecular markers for bovine oocyte competence [46]. During the present study we found the presence of 251 and 261 transcripts of this family in GC of control and heat stressed animals, respectively, however, most of them without significantly different abundance levels (data not shown). To our knowledge, with one exception, members of the solute carrier family have not been published yet in the context of heat stress conditions. Only in a recent study it was found that *SLC5A11* transcripts were up-regulated under acute heat stress conditions in the testes of a broiler-type stain of Taiwan country chicken [47] and was discussed in the context of apoptotic processes. The bovine orthologue of this gene was also found expressed at moderated levels in GC of the HS and PF groups, however without any heat stress related regulation.

A prominently down-regulated transcript under acute heat stress conditions was *BHLHE40* encoding the E-box binding transcription factor “basic helix-loop-helix family, member E40” (Table 3). Expression and regulation of this gene, also known as *SHARP2*, by gonadotropins has been previously studied in rat GC [48]. Members of this family are known to respond to environmental stimuli and regulate several physiological processes in diverse cell types, including cell cycle, apoptosis, and differentiation via their actions as both transcriptional activators and repressors [49]. The physiological role of this factor during folliculogenesis is not yet clear, but its established role as a transcriptional repressor is in line with the observed heat stress induced up-regulation of the vast majority of heat affected transcripts.

Another down-regulated transcript confirmed by qPCR (Table 4) was *MX1* encoding the interferon-inducible GTP-binding protein P78 originally named “myxovirus resistance 1”. For the first time this gene has been described in the context of reproduction in the ovine corpus luteum, where it is induced by conceptus derived interferon tau and showed stabilized expression throughout pregnancy [50]. In this context it was hypothesized that *MX1* might be involved in providing protection or resistance of the corpus luteum to lytic actions and in helping to protect the embryo and early developing fetus from diseases or infections. Interestingly, in mouse ovarian granulosa cells this protein seems involved in the induction of innate antiviral responses [51]. So far however, we have no conclusive explanation for its significant down-regulation in GC of the HS group.

### Acute pre-orulatory heat stress does not specifically affect stress- and apoptosis-related genes in granulosa cells

In the present study, neither heat shock nor stress related, nor apoptosis related genes have been found affected, which is contradictory to several previous studies. In buffalo it was found that COCs recovered during the spring and summer showed significant (P < 0.05) upregulation of HSP70 mRNA expression compared to those collected during the cold season [52]. This suggests that long term exposure to HS throughout the summer in hot countries entails different effects as compared with the present experimental approach of acute pre-ovulatory HS. Heat-induced apoptosis and the involvement of the Bax and BH4 domain dependent pathways was also found when bovine cumulus-oocyte-complexes (COC) were exposed in vitro to 41°C compared to 39°C [53]. Also in another study in cultured sheep GC the colony forming efficiency was decreased and the apoptosis rate was significantly increased under thermal stress. Both effects could be reduced with melatonin supplementation [54]. In pig, culturing of ovarian GC at high (41°C) vs. normal (37.5°C) temperatures resulted in increased abundances of *HSP70*.*2*, *HSP72* and *HSP105/110* mRNAs [55]. In contrast, markers of proliferation and cell cycle as *PCNA* and *cyclin B1* and of apoptosis (Bax, caspase-3) were negatively affected under in vitro heat stress conditions in isolated pig ovarian follicles as well as in cultured GC [56]. Also in a previous in vitro study exposing bovine day 17 conceptuses to heat stress (39°C vs. 43°C) a clear induction of heat shock proteins was found [57]. In a recent study it was also shown in cultured bovine granulosa cells that even mild HS (40.5°C) can induce apoptosis through the BAX/BCL-2 pathway [58]. However, one must be careful to directly compare these contradictory in vitro data with the data of the present in vivo approach. In a previous study it has been shown that the presence of even low serum concentrations induces fundamental changes of the morphological, physiological and molecular properties of cultured bovine granulosa cells leading to a dramatic decrease of the granulosa cell-specific functionality [59].

Interestingly, using an organ cultures approach in pig it was shown that heat shock proteins and levels of the heat shock factor proteins were significantly increased in oocytes whereas the same proteins were completely unaffected in cumulus cells [8]. This was also confirmed by in vivo experiments showing that different heat shock protein and heat shock factor transcripts and proteins were affected in oocytes, but not in cumulus cells of cumulus-oocyte-complexes collected during summer compared to winter [8]. Although the heat stress models and species are different in these studies the results are in accordance with our data. Suggestively, either granulosa derived cells (cumulus, granulosa) are not able to “adequately” respond to heat stress or their response thresholds are different compared to germ line derived or other cell types as also discussed by [8]. An alternative explanation for the absent up-regulation of stress-related genes could be an effective pre-ovulatory temperature gradient that had been observed in several mammalian ovaries with large follicles being 0.5 to 1.5°C cooler than the ovarian stroma [60,61]. This has been found in rabbits and pigs, but might also be present in the bovine. This effect might be caused by heat-consuming processes in the expanding follicullar fluid and by a local transfer of heat between intra-ovarian blood vessels [60]. As a result, the cells within large pre-ovulatory follicles might have been protected from deleterious heat stress under the present experimental conditions.

Accordingly, also due to bioinformatic analysis with the IPA tool specific stress- or heat-related functions or pathways were also not found to be affected. By contrast, more general functions, partly directly related to processes of reproduction were affected (see Fig 3). Altogether 38 different heat affected canonical pathways could be identified by IPA. However, the regulated genes *PIK3C2A*, *PLCD4* and/or *ADCY6* were significantly associated with 24 of them. This suggests that only few basic intracellular pathways as the inositol (IP3/DAG) or cAMP/PKA pathways were affected by the heat stress treatment. Both pathways are part of a plethora of signaling system, mostly associated with G-protein receptors as FSH and LH, or oxytocin signaling. Because all three genes were found up-regulated under HS conditions, this suggests a general rather than specific activation of many intracellular pathways under the present HS conditions. This is also well in line with the observation that nearly 80% of the affected genes were clearly up-regulated. Other frequently involved heat affected genes were *PDGFB* and/or *PDGFC*, encoding “platelet derived growth factor subunit B” and “platelet derived growth factor C”, respectively. These were associated with 12 significantly affected pathways (see S4 Table). This is well in line with the -established role of the platelet derived growth factor pathway to control steroidogenesis in both sexes thus representing a conserved mechanism in the local control of steroidogenic cell lineages [62].

## Conclusions

From these data we conclude that acute pre-ovulatory heat stress does not affect specific stress- or apoptosis-related, but instead more general intracellular signaling pathways in granulosa cells. The data also provide first evidence that heat induced alterations of the activin-inhibin-follistatin system might in part be responsible for the possibly compromised growth of the dominant follicle.

## Supporting Information

S1 Table Primers used for quantitative real-time PCR (qPCR). (DOCX)

S2 Table Transcript abundance of different housekeeping genes in samples of the HS and PF groups. (DOCX)

S3 Table List of differentially expressed transcripts between samples of the HS and PF group. (DOCX)

S4 Table Significantly affected Canonical Pathways according to IPA analysis. (DOCX)

## Abbreviations

BW: body weight
d: day(s)
(m)GC: (mural) granulosa cells
E2: estradiol
FC: fold change
GnRH: gonadotropin releasing hormone
h: hour(s)
HP: metabolic heat production
HS: heat stress
LH: luteinizing hormone
mBW: metabolic body weight
NEB: negative energy balance
P4: progesterone
PF: pair-fed
PGF_2α_: Prostaglandin F2α
RIA: radioimmunoassay
THI: Temperature Humidity Index
