# Supplementary material for: Exposure of Lactating Dairy Cows to Acute Pre-Ovulatory Heat Stress Affects Granulosa Cell-Specific Gene Expression Profiles in Dominant Follicles
Source: PLoS One. 2016 Aug 17;11(8):e0160600. doi: 10.1371/journal.pone.0160600 (PMC4988698; doi:10.1371/journal.pone.0160600)
Supplement: S1 Table — (DOCX) [file pone.0160600.s001.docx]

S1 Table. Primers used for quantitative real-time PCR (qPCR)

| Gene Symbol |  | Sequence | Size (bp) | Accession No. |
| --- | --- | --- | --- | --- |
| *ADCY6* | for | TCCCCTGTGTGAGGGCACCGC | 233 | NM_001143877 |
|  | rev | TGGAAGAAGCCAAGCCGTGGACGC |  |  |
| *BHLHE40* | for | AGCGGATCCCCAGCGCGCAA | 153 | NM_001024929 |
|  | rev | AGGTCTCCTTGCTGTCCTCGCTCCG |  |  |
| *B2M* | for | ACGCTGAGTTCACTCCCAACAGCAA | 114 | NM_173893 |
|  | rev | TCGATGGTGCTGCTTACAGGTCTCG |  |  |
| *CRHBP* | for | GCCGACCGGCCGCAGCTACA | 189 | NM_001101202 |
|  | rev | CCTCTCCGTCGTGGGGAGAGGGT |  |  |
| *FST* | for | GGCGCCCCCAACTGCATCCCT | 158 | NM_175801 |
|  | rev | CCATCCAGCCCACACACCGGGC |  |  |
| *GAPDH* | for | AGCGAGATCCTGCCAACATCAAG | 221 | NM_001034034 |
|  | rev | GCAGGAGGCATTGCTGACAATCT |  |  |
| *GK* | for | GGCTCCGCGCCGGTAAAGGGG | 139 | NM_001075236 |
|  | rev | GGCGGGCGGTCTTGACCCGT |  |  |
| *GNG12* | for | TGCCCAGGCACGGAGGACTGT | 131 | NM_174785 |
|  | rev | CCATCAGCAGAGGGTCATTCCTGGC |  |  |
| *HAS3* | for | GTGGGGAGGCAAGCGAGAGG | 177 | NM_001192867 |
|  | rev | AGGATTTGGACATCTCCCCCGAC |  |  |
| *HMBS* | for | CAGCATGAAGATGGCCCTGAAGATG | 249 | NM_001046207 |
|  | rev | CTCAGGTAGCAGAGGGCTGGGATGT |  |  |
| *HPRT1* | for | TGAAAAGGACCCCTCGAAGTGTTGG | 200 | NM_001034035 |
|  | rev | CGCCAGGTATTTCCAAACTCAACTCG |  |  |
| *IFI47* | for | GCGCTACACCGCTCTCCTCTTGTTGC | 117 | NM_001034545 |
|  | rev | AAGGCCAAAATGGCTGCCCGGAAGG |  |  |
| *IFIT1* | for | TGGGCCAACAGGCAGAAGCCCAG | 249 | XM_002698357 |
|  | rev | AGCCCTCCAGGCGATAGACAACGA |  |  |
| *MAP2K1* | for | CATGTCGCCAGAGCGACTCCAGGG | 190 | NM_001130752 |
|  | rev | GGTCCTTGGCCTGGGCGGGG |  |  |
| *MX1* | for | GGGACGGGCGGTGTTGGAGCA | 250 | NM_173940 |
|  | rev | ACGCCCAGGGACCGCAGGGA |  |  |
| *OAS1Y* | for | TTCTGGACCCGGCGGACCCT | 153 | NM_001040606 |
|  | rev | CTTGGGGCGACACATCCCAGGAGC |  |  |
| *PDGFC* | for | CCGTCCTCGCTGCCCGCCTC | 209 | XM_002694441 |
|  | rev | AGCAGGTTCAGGTCCACCACTCTGG |  |  |
| *PIK3C2A* | for | GGCAGAGATCGAGGCATGGTGGAGC | 118? | XM_001787474 |
|  | rev | TCCTCAGCCACTCAGCAAGCGGT |  |  |
| *PLCD4* | for | TGCATGCAGCAAGGTTACCGCCAC | 141 | NM_001046489 |
|  | rev | TCCGCCTGTGAAGTGGCTGCCT |  |  |
| *PRKACB* | for | TCACCCCTTCCTCCCATCGCCCC | 80 | NM_174585 |
|  | rev | TCTTTCACGCTCTCCACCTCGCTGC |  |  |
| *PRKD1* | for | GGACACGCTGCGGAAGCGGC | 241 | XM_612625 |
|  | rev | TGGTGGCGGGCTGGAGGGGT |  |  |
| *RPLP0* | for | TGGTTACCCAACCGTCGCATCTGTA | 142 | NM_001012682 |
|  | rev | CACAAAGGCAGATGGATCAGCCAAG |  |  |
| *RPS18* | for | GAGGTGGAACGTGTGATCACCATT | 100 | NM_001033614 |
|  | rev | TGTATTTCCCGTCCTTCACGTCCT |  |  |
| *SLC27A1* | for | AGCCCACGACCGAGCAGCCAA | 144 | NM_001033625 |
|  | rev | CCCGGAGCTCGCATCCTAGAGACCC |  |  |
| *TBP* | for | GCCTTGTGCTTACCCACCAACAGTTC | 200 | NM_001075742 |
|  | rev | TGTCTTCCTGAAACCCTTCAGAATAGGG |  |  |
