# Supplementary material for: Exposure of Lactating Dairy Cows to Acute Pre-Ovulatory Heat Stress Affects Granulosa Cell-Specific Gene Expression Profiles in Dominant Follicles
Source: PLoS One. 2016 Aug 17;11(8):e0160600. doi: 10.1371/journal.pone.0160600 (PMC4988698; doi:10.1371/journal.pone.0160600)
Supplement: S2 Table — (DOCX) [file pone.0160600.s002.docx]

S2 Table. Transcript abundance of different housekeeping genes in samples of the HS and PF groups.

|  | RPS18 | RPLP0 | B2M | GAPDH | HPRT1 | TBP | HMBS |
| --- | --- | --- | --- | --- | --- | --- | --- |
| HS | 1.4E+08 | 1.2E+08 | 4.9E+07 | 1.8E+08 | 1.5E+07 | 2.8E+06 | 1.5E+06 |
|  | ±1.4E+07 | ±9.1E+06 | ±1.3E+07 | ±3.2E+07 | ±3.0E+06 | ±2.6E+05 | ±1.7E+05 |
| PF | 1.8E+08 | 1.5E+08 | 9.6E+07 | 9.6E+07 | 7.3E+06 | 2.3E+06 | 1.1E+06 |
|  | ±2.7E+07 | ±2.8E+07 | ±2.9E+07 | ±2.7E+07 | ±2.3E+06 | ±1.9E+05 | ±1.4E+05 |
|  |  |  |  |  |  |  |  |
| FC | -1.3 | -1.2 | -2.0 | 1.9 | 2.0 | 1.2 | 1.4 |
| p | 0.24 | 0.39 | 0.18 | 0.08 | 0.10 | 0.16 | 0.10 |

Values are means of absolute abundance [copy numbers/µg RNA] ± SEM. FC, fold changes; p, p-values from unpaired t-tests comparing means from the HS and PF groups.
