# Supplementary material for: Exposure of Lactating Dairy Cows to Acute Pre-Ovulatory Heat Stress Affects Granulosa Cell-Specific Gene Expression Profiles in Dominant Follicles
Source: PLoS One. 2016 Aug 17;11(8):e0160600. doi: 10.1371/journal.pone.0160600 (PMC4988698; doi:10.1371/journal.pone.0160600)
Supplement: S3 Table — (DOCX) [file pone.0160600.s003.docx]

S3 Table. List of differentially expressed transcripts between samples of the HS and PF group.

| **Transcript Cluster ID** | **Gene Symbol** | **HS Signal** | **PF Signal** | **Fold Change** | **p-value** | **Description** |
| --- | --- | --- | --- | --- | --- | --- |
| 12749590 | HAS3 | **617.4** | **116.2** | 5.35 | 0.019 | hyaluronan synthase 3 |
| 12914061 |  | **18.0** | **4.1** | 4.36 | 0.026 |  |
| 12737098 | B3GALT2 | **177.3** | **43.7** | 4.06 | 0.002 | UDP-Gal:betaGlcNAc beta 1,3-galactosyltransferase, polypeptide 2 |
| 12884128 | CYP4F2 | **190.0** | **48.5** | 3.93 | 0.010 | cytochrome P450, family 4, subfamily F, polypeptide 2 |
| 12872484 | SLC9B2 | **584.1** | **153.3** | 3.81 | 0.006 | solute carrier family 9, subfamily B (cation proton antiporter 2), member 2 |
| 12844361 | KCNJ13 | **81.6** | **21.9** | 3.73 | 0.003 | potassium inwardly-rectifying channel, subfamily J, member 13 |
| 12899216 | MIR2480 | **15.3** | **4.1** | 3.70 | 0.004 | microRNA mir-2480 |
| 12894406 | ADAMTSL1 | **415.9** | **113.0** | 3.66 | 0.033 | ADAMTS-like 1 |
| 12809501 | CABYR | **90.5** | **25.6** | 3.53 | 0.019 | calcium binding tyrosine-(Y)-phosphorylation regulated |
| 12691248 | CRHBP | **268.7** | **77.2** | 3.46 | 0.009 | corticotropin releasing hormone binding protein |
| 12908260 | CA5B | **512.0** | **153.3** | 3.35 | 0.030 | carbonic anhydrase VB, mitochondrial |
| 12897800 | WASF1 | **568.1** | **170.1** | 3.34 | 0.029 | WAS protein family, member 1 |
| 12757255 | SMPD3 | **148.1** | **48.2** | 3.08 | 0.014 | sphingomyelin phosphodiesterase 3, neutral membrane (neutral sphingomyelinase II) |
| 12898611 | HS3ST5 | **167.7** | **58.1** | 2.88 | 0.026 | heparan sulfate (glucosamine) 3-O-sulfotransferase 5 |
| 12847375 | CDC14A | **494.6** | **173.6** | 2.85 | 0.005 | CDC14 cell division cycle 14 homolog A (S. cerevisiae) |
| 12821015 | SLC35G1 | **284.0** | **99.7** | 2.85 | 0.013 | solute carrier family 35, member G1 |
| 12808949 |  | **121.1** | **44.0** | 2.75 | 0.003 |  |
| 12778973 | PLCD4 | **168.9** | **61.8** | 2.73 | 0.005 | phospholipase C, delta 4 |
| 12903581 | ACE2 | **760.1** | **278.2** | 2.72 | 0.024 | angiotensin I converting enzyme (peptidyl-dipeptidase A) 2 |
| 12805918 | RBM24 | **388.0** | **143.0** | 2.71 | 0.030 | RNA binding motif protein 24 |
| 12908846 | GABRE | **121.1** | **44.9** | 2.70 | 0.000 | gamma-aminobutyric acid (GABA) A receptor, epsilon |
| 12907479 | GK | **196.7** | **73.0** | 2.69 | 0.017 | glycerol kinase |
| 12707468 | GPR75 | **159.8** | **59.7** | 2.68 | 0.001 | G protein-coupled receptor 75 |
| 12895096 | TRPM3 | **110.7** | **41.6** | 2.66 | 0.001 | transient receptor potential cation channel, subfamily M, member 3 |
| 12892532 | ADAMTSL1 | **286.0** | **107.6** | 2.65 | 0.029 | ADAMTS-like 1 |
| 12739086 | SLC30A10 | **101.8** | **38.6** | 2.63 | 0.000 | solute carrier family 30, member 10 |
| 12816534 | STYXL1 | **134.4** | **51.6** | 2.61 | 0.001 | serine/threonine/tyrosine interacting-like 1 |
| 12765856 | SECTM1 | **164.3** | **63.1** | 2.60 | 0.046 | secreted and transmembrane 1 |
| 12810182 | TTC39C | **670.9** | **259.6** | 2.59 | 0.033 | tetratricopeptide repeat protein 39C-like |
| 12706420 | TBC1D8 | **552.6** | **218.3** | 2.54 | 0.020 | TBC1 domain family, member 8 (with GRAM domain) |
| 12725324 | CA8 | **143.0** | **56.9** | 2.52 | 0.020 | carbonic anhydrase VIII |
| 12777467 | CCDC141 | **91.8** | **36.5** | 2.52 | 0.007 | coiled-coil domain containing 141 |
| 12728656 | PLEKHA7 | **194.0** | **78.2** | 2.48 | 0.026 | pleckstrin homology domain containing, family A member 7 |
| 12686973 | TFF2 | **57.3** | **23.6** | 2.43 | 0.011 | trefoil factor 2 |
| 12720083 | CAMK1D | **184.8** | **76.1** | 2.42 | 0.039 | calcium/calmodulin-dependent protein kinase ID |
| 12861966 | NR1H4 | **41.1** | **17.0** | 2.42 | 0.009 | nuclear receptor subfamily 1, group H, member 4 |
| 12702344 | CYP26B1 | **106.2** | **44.3** | 2.39 | 0.026 | cytochrome P450, family 26, subfamily B, polypeptide 1 |
| 12708482 | LOC783253 | **8.8** | **3.7** | 2.39 | 0.000 | FCF1 small subunit-like |
| 12890086 | EPHX2 | **461.4** | **198.1** | 2.34 | 0.021 | epoxide hydrolase 2, cytoplasmic |
| 12894119 | PTCH1 | **564.2** | **243.9** | 2.32 | 0.037 | patched 1 |
| 12853791 | FAM115C | **100.4** | **44.0** | 2.28 | 0.014 | family with sequence similarity 115, member C |
| 12803236 | FAM65B | **286.0** | **125.4** | 2.28 | 0.034 | family with sequence similarity 65, member B |
| 12883870 | SLC5A5 | **89.3** | **39.4** | 2.26 | 0.018 | solute carrier family 5 (sodium iodide symporter), member 5 |
| 12912033 |  | **9.3** | **4.1** | 2.25 | 0.018 |  |
| 12712609 | KBTBD7 | **80.4** | **36.8** | 2.20 | 0.034 | kelch repeat and BTB (POZ) domain containing 7 |
| 12858784 | ADCY6 | **519.1** | **238.9** | 2.18 | 0.032 | adenylate cyclase 6 |
| 12864109 | CACNB3 | **508.5** | **233.9** | 2.18 | 0.036 | calcium channel, voltage-dependent, beta 3 subunit |
| 12787048 | FST | **1509.7** | **689.8** | 2.18 | 0.041 | follistatin |
| 12886857 | CYP4F3 | **49.5** | **22.8** | 2.17 | 0.024 | cytochrome P450, family 4, subfamily F, polypeptide 3 |
| 12729339 | PIK3C2A | **630.3** | **290.0** | 2.17 | 0.005 | phosphatidylinositol-4-phosphate 3-kinase, catalytic subunit type 2 alpha |
| 12739614 | PROX1 | **319.6** | **148.1** | 2.17 | 0.044 | prospero homeobox 1 |
| 12844142 | SLC27A3 | **80.4** | **37.3** | 2.17 | 0.049 | solute carrier family 27 (fatty acid transporter), member 3 |
| 12828783 | RHOBTB1 | **1168.1** | **548.7** | 2.14 | 0.041 | Rho-related BTB domain containing 1 |
| 12836295 | TPM3 | **215.3** | **102.5** | 2.10 | 0.019 | tropomyosin 3 |
| 12885340 | SLC27A1 | **172.4** | **82.7** | 2.09 | 0.027 | solute carrier family 27 (fatty acid transporter), member 1 |
| 12724021 |  | **9.8** | **4.8** | 2.06 | 0.024 |  |
| 12858199 | LRIG3 | **552.6** | **270.6** | 2.05 | 0.031 | leucine-rich repeats and immunoglobulin-like domains 3 |
| 12904842 | SLC9A6 | **221.3** | **107.6** | 2.05 | 0.003 | solute carrier family 9 (sodium/hydrogen exchanger), member 6 |
| 12686044 | LOC537248 | **115.4** | **56.5** | 2.04 | 0.006 | acid phosphatase-like protein 2-like |
| 12809800 | TAF4B | **108.4** | **53.4** | 2.04 | 0.002 | TAF4b RNA polymerase II, TATA box binding protein (TBP)-associated factor, 105kDa |
| 12824413 | TRAPPC11 | **415.9** | **203.7** | 2.04 | 0.012 | trafficking protein particle complex 11 |
| 12807325 | CEP192 | **245.6** | **120.3** | 2.03 | 0.010 | centrosomal protein 192kDa |
| 12914389 |  | **19.4** | **9.6** | 2.03 | 0.039 |  |
| 12906109 | DDX26B | **284.0** | **142.0** | 2.01 | 0.041 |  |
| 12845155 |  | **65.8** | **32.9** | 2.00 | 0.011 |  |
| 12911743 |  | **25.5** | **12.7** | 2.00 | 0.014 |  |
| 12780755 | RCAN3 | **196.7** | **99.0** | 1.99 | 0.041 | RCAN family member 3 |
| 12893324 | ALAD | **326.3** | **165.4** | 1.98 | 0.035 | aminolevulinate dehydratase |
| 12906577 | MIR224 | **7.4** | **3.7** | 1.98 | 0.010 | microRNA mir-224 |
| 12719938 | SAMHD1 | **604.7** | **304.4** | 1.98 | 0.019 | SAM domain and HD domain 1 |
| 12892548 | TRPM3 | **38.1** | **19.4** | 1.96 | 0.004 | transient receptor potential cation channel, subfamily M, member 3 |
| 12733269 | PGAP2 | **1260.7** | **648.1** | 1.95 | 0.027 | post-GPI attachment to proteins 2 |
| 12895464 | C8H9orf84 | **18.3** | **9.5** | 1.92 | 0.028 | chromosome 8 open reading frame, human C9orf84 |
| 12809729 | CEP76 | **296.1** | **154.3** | 1.92 | 0.008 | centrosomal protein 76kDa |
| 12823595 | ING2 | **916.5** | **477.7** | 1.92 | 0.033 | inhibitor of growth family, member 2 |
| 12692884 | RNASE10 | **10.3** | **5.4** | 1.92 | 0.012 | ribonuclease, RNase A family, 10 (non-active) |
| 12825006 | FAT1 | **1478.6** | **776.0** | 1.91 | 0.021 | FAT tumor suppressor homolog 1 (Drosophila) |
| 12902272 | MAMLD1 | **349.7** | **183.5** | 1.91 | 0.017 |  |
| 12883021 | PRELID2 | **30.3** | **15.9** | 1.91 | 0.015 | PRELI domain containing 2 |
| 12690482 | MAP2K1 | **198.1** | **104.0** | 1.90 | 0.025 | mitogen-activated protein kinase kinase 1 |
| 12912199 |  | **66.7** | **35.3** | 1.90 | 0.002 |  |
| 12890715 | GLIS3 | **64.9** | **34.1** | 1.89 | 0.037 |  |
| 12914339 |  | **50.6** | **26.9** | 1.89 | 0.019 |  |
| 12804265 | MIR877 | **21.9** | **11.6** | 1.88 | 0.014 | microRNA mir-877 |
| 12779057 | NABP1 | **337.8** | **179.8** | 1.88 | 0.011 | oligonucleotide/oligosaccharide-binding fold containing 2A |
| 12842793 | PRKACB | **657.1** | **349.7** | 1.88 | 0.011 | protein kinase, cAMP-dependent, catalytic, beta |
| 12795280 | ZFYVE20 | **436.5** | **232.3** | 1.88 | 0.001 | zinc finger, FYVE domain containing 20 |
| 12754372 | KLHL36 | **153.3** | **82.1** | 1.86 | 0.017 | kelch-like 36 (Drosophila) |
| 12915197 |  | **284.0** | **153.3** | 1.86 | 0.044 |  |
| 12870891 | CCNG2 | **167.7** | **90.5** | 1.85 | 0.009 | cyclin G2 |
| 12686832 | MRPS6 | **786.9** | **424.6** | 1.85 | 0.043 | mitochondrial ribosomal protein S6 |
| 12800795 | NBEAL2 | **141.0** | **76.1** | 1.85 | 0.029 | neurobeachin-like 2 |
| 12681903 | TNIK | **171.3** | **92.4** | 1.85 | 0.042 | TRAF2 and NCK interacting kinase |
| 12850342 | TNS3 | **680.3** | **369.6** | 1.84 | 0.011 | tensin 3 |
| 12793185 | PRKD1 | **749.6** | **410.1** | 1.83 | 0.029 | protein kinase D1 |
| 12909023 | ATP11C | **560.3** | **315.2** | 1.78 | 0.021 | ATPase, class VI, type 11C |
| 12776378 | PTPN4 | **229.1** | **128.9** | 1.78 | 0.035 | protein tyrosine phosphatase, non-receptor type 4 (megakaryocyte) |
| 12723600 | SYBU | **31.8** | **17.9** | 1.77 | 0.014 | syntabulin (syntaxin-interacting) |
| 12723492 | TSPYL5 | **410.1** | **232.3** | 1.77 | 0.009 | TSPY-like 5 |
| 12871419 | RASL11B | **202.3** | **115.4** | 1.76 | 0.017 | RAS-like, family 11, member B |
| 12795739 | HYAL1 | **128.9** | **74.0** | 1.75 | 0.012 | hyaluronoglucosaminidase 1 |
| 12864750 | LARGE | **451.9** | **259.6** | 1.75 | 0.010 | like-glycosyltransferase |
| 12905931 |  | **34.1** | **19.6** | 1.75 | 0.007 |  |
| 12852561 |  | **46.2** | **26.5** | 1.75 | 0.008 |  |
| 12742365 | CAMKK2 | **666.3** | **382.7** | 1.74 | 0.042 | calcium/calmodulin-dependent protein kinase kinase 2, beta |
| 12915425 |  | **515.6** | **296.1** | 1.74 | 0.029 |  |
| 12905449 | KLHL15 | **685.0** | **393.4** | 1.73 | 0.023 | kelch-like 15 (Drosophila) |
| 12723771 | SAMD12 | **66.7** | **38.6** | 1.73 | 0.047 | sterile alpha motif domain containing 12 |
| 12770498 | TIMM22 | **149.1** | **86.8** | 1.73 | 0.046 | translocase of inner mitochondrial membrane 22 homolog (yeast) |
| 12909853 | GPC4 | **132.5** | **77.2** | 1.72 | 0.010 | glypican 4 |
| 12798637 | OSBPL10 | **347.3** | **200.9** | 1.72 | 0.046 | oxysterol binding protein-like 10 |
| 12914089 |  | **51.3** | **29.9** | 1.72 | 0.042 |  |
| 12763396 | ABR | **770.7** | **451.9** | 1.71 | 0.036 | active BCR-related gene |
| 12753878 | CCDC8 | **302.3** | **176.1** | 1.71 | 0.029 | coiled-coil domain containing 8 |
| 12914949 |  | **505.0** | **296.1** | 1.70 | 0.037 |  |
| 12694790 | CDO1 | **576.0** | **340.1** | 1.69 | 0.042 | cysteine dioxygenase, type I |
| 12860640 | CHST11 | **968.8** | **576.0** | 1.68 | 0.040 | carbohydrate (chondroitin 4) sulfotransferase 11 |
| 12798341 | CRELD1 | **106.9** | **63.6** | 1.68 | 0.049 | cysteine-rich with EGF-like domains 1 |
| 12860403 | CSDC2 | **109.9** | **65.3** | 1.68 | 0.003 | cold shock domain containing C2, RNA binding |
| 12852752 | MIR2284B | **5.0** | **2.9** | 1.68 | 0.035 | microRNA mir-2284b |
| 12890269 | UGCG | **158.7** | **93.7** | 1.68 | 0.009 | UDP-glucose ceramide glucosyltransferase |
| 12873335 | ATP8A1 | **119.4** | **72.0** | 1.67 | 0.005 | ATPase, aminophospholipid transporter (APLT), class I, type 8A, member 1 |
| 12851128 | KLHL7 | **106.2** | **63.6** | 1.67 | 0.008 | kelch-like 7 (Drosophila) |
| 12769077 | RNF213 | **564.2** | **340.1** | 1.67 | 0.008 | ring finger protein 213; ring finger protein 213-like |
| 12900030 | ARHGAP18 | **398.9** | **242.2** | 1.66 | 0.001 | Rho GTPase activating protein 18 |
| 12747734 | EP400 | **354.6** | **213.8** | 1.66 | 0.027 | E1A binding protein p400 |
| 12682459 | GK5 | **136.2** | **82.1** | 1.66 | 0.015 | glycerol kinase 5 (putative) |
| 12784378 | NIPAL3 | **975.5** | **588.1** | 1.66 | 0.034 | NIPA-like domain containing 3 |
| 12807909 | EMILIN2 | **177.3** | **107.6** | 1.65 | 0.041 | elastin microfibril interfacer 2 |
| 12681420 | XXYLT1 | **306.6** | **184.8** | 1.65 | 0.014 | xyloside xylosyltransferase 1 |
| 12900172 | CDC40 | **207.9** | **126.2** | 1.64 | 0.029 | cell division cycle 40 homolog (S. cerevisiae) |
| 12815958 | EMP2 | **1097.5** | **670.9** | 1.64 | 0.005 | epithelial membrane protein 2 |
| 12808817 | KCTD1 | **49.2** | **30.1** | 1.64 | 0.010 | potassium channel tetramerisation domain containing 1 |
| 12896459 | PLEKHG1 | **580.0** | **354.6** | 1.64 | 0.037 | pleckstrin homology domain containing, family G (with RhoGef domain) member 1 |
| 12910712 | SCML1 | **74.5** | **45.6** | 1.64 | 0.038 |  |
| 12833243 | SIAE | **344.9** | **209.4** | 1.64 | 0.046 | sialic acid acetylesterase |
| 12834341 | SLC29A2 | **144.0** | **88.0** | 1.64 | 0.045 | solute carrier family 29 (nucleoside transporters), member 2 |
| 12686419 | UBXN7 | **1105.1** | **675.6** | 1.64 | 0.003 | UBX domain protein 7 |
| 12732636 | ARNTL | **95.0** | **58.5** | 1.63 | 0.012 | aryl hydrocarbon receptor nuclear translocator-like |
| 12686491 | BRWD1 | **278.2** | **170.1** | 1.63 | 0.034 | bromodomain and WD repeat domain containing 1 |
| 12755271 | C18H19orf18 | **26.9** | **16.4** | 1.63 | 0.015 | chromosome 18 open reading frame, human C19orf18 |
| 12703362 | FAM228B | **21.0** | **12.8** | 1.63 | 0.018 | family with sequence similarity 228, member B |
| 12891144 | KIF13B | **263.2** | **162.0** | 1.63 | 0.048 | kinesin family member 13B |
| 12821454 | NT5C2 | **276.3** | **168.9** | 1.63 | 0.007 | 5'-nucleotidase, cytosolic II |
| 12780866 | TANC1 | **427.6** | **263.2** | 1.63 | 0.039 | tetratricopeptide repeat, ankyrin repeat and coiled-coil containing 1 |
| 12914571 |  | **77.2** | **47.5** | 1.63 | 0.031 |  |
| 12746214 | ARHGAP10 | **617.4** | **382.7** | 1.62 | 0.008 | Rho GTPase activating protein 10 |
| 12892265 | ERCC6L2 | **64.0** | **39.7** | 1.62 | 0.022 | excision repair cross-complementing rodent repair deficiency, compl. group 6-like 2 |
| 12724318 | MYBL1 | **71.5** | **44.3** | 1.62 | 0.047 | v-myb myeloblastosis viral oncogene homolog (avian)-like 1 |
| 12690714 | ZWILCH | **229.1** | **142.0** | 1.62 | 0.013 | Zwilch, kinetochore associated, homolog (Drosophila) |
| 12719162 | EDEM2 | **519.1** | **321.8** | 1.61 | 0.049 | ER degradation enhancer, mannosidase alpha-like 2 |
| 12780661 | GPD2 | **76.1** | **47.2** | 1.61 | 0.018 | glycerol-3-phosphate dehydrogenase 2 (mitochondrial) |
| 12802652 | PAQR8 | **61.4** | **38.1** | 1.61 | 0.044 | progestin and adipoQ receptor family member VIII |
| 12841781 | NUP210L | **46.9** | **29.2** | 1.60 | 0.005 |  |
| 12808583 | SS18 | **537.5** | **335.5** | 1.60 | 0.006 | synovial sarcoma translocation, chromosome 18 |
| 12738151 | BT.88134 | **65.8** | **41.4** | 1.59 | 0.025 |  |
| 12859771 | DENND5B | **352.1** | **221.3** | 1.59 | 0.010 | DENN/MADD domain containing 5B |
| 12782069 | NOSTRIN | **359.5** | **224.4** | 1.59 | 0.003 | nitric oxide synthase trafficker |
| 12713650 | PLCB4 | **1074.9** | **675.6** | 1.59 | 0.005 | phospholipase C, beta 4 |
| 12679622 | WRB | **296.1** | **187.4** | 1.59 | 0.004 | tryptophan rich basic protein |
| 12697892 | BT.73361 | **52.3** | **33.1** | 1.58 | 0.045 |  |
| 12752956 | MED25 | **760.1** | **481.0** | 1.58 | 0.036 | mediator complex subunit 25 |
| 12883433 | SQSTM1 | **1089.9** | **685.0** | 1.58 | 0.033 | sequestosome 1 |
| 12912059 |  | **24.1** | **15.2** | 1.58 | 0.032 |  |
| 12908922 | ACRC | **65.8** | **41.9** | 1.57 | 0.042 | acidic repeat containing |
| 12729627 | LOC540128 | **4.6** | **2.9** | 1.57 | 0.032 | olfactory receptor, family 4, subfamily X, member 2-like |
| 12866871 | MICAL3 | **99.7** | **63.6** | 1.57 | 0.019 | microtubule associated monoxygenase, calponin and LIM domain containing 3 |
| 12846121 | NPR1 | **165.4** | **105.4** | 1.57 | 0.032 | natriuretic peptide receptor A/guanylate cyclase A (atrionatriuretic peptide receptor A) |
| 12906034 |  | **5.7** | **3.6** | 1.57 | 0.022 |  |
| 12759486 | CHTF8 | **1341.8** | **855.1** | 1.56 | 0.043 | CTF8, chromosome transmission fidelity factor 8 homolog (S. cerevisiae) |
| 12861924 | KANSL2 | **372.2** | **238.9** | 1.56 | 0.004 | KAT8 regulatory NSL complex subunit 2 |
| 12702509 | NPAS2 | **113.0** | **72.5** | 1.56 | 0.017 | neuronal PAS domain protein 2 |
| 12766339 | PRPSAP1 | **377.4** | **242.2** | 1.56 | 0.011 | phosphoribosyl pyrophosphate synthetase-associated protein 1 |
| 12873002 | USP53 | **770.7** | **494.6** | 1.56 | 0.025 | ubiquitin specific peptidase 53 |
| 12714853 | CRLS1 | **340.1** | **218.3** | 1.55 | 0.045 | cardiolipin synthase 1 |
| 12769517 | GJC1 | **90.5** | **58.5** | 1.55 | 0.039 | gap junction protein, gamma 1, 45kDa |
| 12904630 | HTATSF1 | **337.8** | **216.8** | 1.55 | 0.008 | HIV-1 Tat specific factor 1 |
| 12897282 | REV3L | **421.7** | **270.6** | 1.55 | 0.040 | REV3-like, catalytic subunit of DNA polymerase zeta (yeast) |
| 12835758 | SV2A | **67.2** | **43.1** | 1.55 | 0.028 | synaptic vesicle glycoprotein 2A |
| 12832185 | TTC9C | **124.5** | **80.4** | 1.55 | 0.040 | tetratricopeptide repeat domain 9C |
| 12789821 | UBE2Q2 | **265.0** | **172.4** | 1.55 | 0.009 | ubiquitin-conjugating enzyme E2Q family member 2 |
| 12914825 |  | **46.5** | **30.1** | 1.55 | 0.043 |  |
| 12793978 | C21H15orf39 | **113.8** | **73.5** | 1.54 | 0.016 | chromosome 21 open reading frame, human C15orf39 |
| 12785671 | CMBL | **227.5** | **147.0** | 1.54 | 0.039 | carboxymethylenebutenolidase homolog (Pseudomonas) |
| 12709373 | FNDC3A | **699.4** | **455.1** | 1.54 | 0.047 | fibronectin type III domain containing 3A |
| 12795511 | LOC514296 | **996.0** | **648.1** | 1.54 | 0.011 | 5'-nucleotidase domain-containing protein 2-like |
| 12733942 | LOC785899 | **18.6** | **12.0** | 1.54 | 0.013 | olfactory receptor, family 5, subfamily AN, member 1-like |
| 12885292 | MYO9B | **240.5** | **155.4** | 1.54 | 0.027 | myosin IXB |
| 12695057 | NYNRIN | **235.6** | **152.2** | 1.54 | 0.000 | NYN domain and retroviral integrase containing |
| 12860149 | SLC11A2 | **324.0** | **210.8** | 1.54 | 0.020 | solute carrier family 11 (proton-coupled divalent metal ion transporters), member 2 |
| 12793904 | SNRPN; SNRPB; SNURF | **199.5** | **129.8** | 1.54 | 0.021 | small nuclear ribonucleoprotein polypeptide N; small nuclear ribonucleoprotein polypeptides B and B1; SNRPN upstream reading frame |
| 12795122 |  | **33.4** | **21.7** | 1.54 | 0.001 |  |
| 12843338 | AHCYL1 | **621.7** | **407.3** | 1.53 | 0.002 | adenosylhomocysteinase-like 1 |
| 12694170 | C10H14orf37 | **218.3** | **142.0** | 1.53 | 0.039 | chromosome 10 open reading frame, human C14orf37 |
| 12710288 | DGKH | **298.2** | **195.4** | 1.53 | 0.036 | diacylglycerol kinase, eta |
| 12690344 | JDP2 | **116.2** | **76.1** | 1.53 | 0.007 | Jun dimerization protein 2 |
| 12748642 | LOC100139764 | **66.3** | **43.1** | 1.53 | 0.004 | WW domain-binding protein 4-like |
| 12824387 | LOC535434 | **99.7** | **65.3** | 1.53 | 0.045 | storkhead-box protein 2-like |
| 12707198 | LPIN1 | **100.4** | **65.8** | 1.53 | 0.046 | lipin 1 |
| 12797808 | MIR2370 | **17.6** | **11.5** | 1.53 | 0.029 | microRNA mir-2370 |
| 12699741 | RNF103 | **300.2** | **196.7** | 1.53 | 0.005 | ring finger protein 103 |
| 12709691 | TM9SF2 | **1937.5** | **1260.7** | 1.53 | 0.015 | transmembrane 9 superfamily member 2 |
| 12815058 | CPPED1 | **52.3** | **34.3** | 1.52 | 0.009 | calcineurin-like phosphoesterase domain containing 1 |
| 12796582 | FAM116A | **229.1** | **151.2** | 1.52 | 0.017 | family with sequence similarity 116, member A |
| 12870678 | FAM47E | **109.9** | **72.0** | 1.52 | 0.042 | family with sequence similarity 47, member E |
| 12683496 | ITGB5 | **1370.0** | **903.9** | 1.52 | 0.041 | integrin, beta 5 |
| 12691299 | KATNBL1 | **195.4** | **128.0** | 1.52 | 0.024 | katanin p80 subunit B-like 1 |
| 12802096 | KCTD20 | **433.5** | **286.0** | 1.52 | 0.037 | potassium channel tetramerisation domain containing 20 |
| 12841721 | KIAA0319L | **280.1** | **184.8** | 1.52 | 0.020 | KIAA0319-like ortholog |
| 12823387 | LOC100296401 | **8.0** | **5.3** | 1.52 | 0.025 | U6 snRNA-associated Sm-like protein LSm5-like |
| 12706700 | AAK1 | **809.0** | **533.7** | 1.51 | 0.014 | AP2 associated kinase 1 |
| 12712601 | POLR1D | **83.9** | **55.7** | 1.51 | 0.040 | polymerase (RNA) I polypeptide D, 16kDa |
| 12837997 | PRKAB2 | **436.5** | **290.0** | 1.51 | 0.035 | protein kinase, AMP-activated, beta 2 non-catalytic subunit |
| 12736373 | SLC35E2 | **219.8** | **145.0** | 1.51 | 0.026 | solute carrier family 35, member E2 |
| 12750768 | USP10 | **704.3** | **464.6** | 1.51 | 0.022 | ubiquitin specific peptidase 10 |
| 12840065 | VPS72 | **501.5** | **333.1** | 1.51 | 0.016 | vacuolar protein sorting 72 homolog (S. cerevisiae) |
| 12913439 |  | **11.0** | **7.3** | 1.51 | 0.014 |  |
| 12745384 | CRYBB1 | **42.2** | **64.0** | -1.51 | 0.036 | crystallin, beta B1 |
| 12693121 | LOC100298572 | **45.9** | **69.1** | -1.51 | 0.013 | uncharacterized LOC100298572 |
| 12789196 | LOC100300716 | **7.0** | **10.6** | -1.51 | 0.006 | uncharacterized LOC100300716 |
| 12911199 |  | **349.7** | **530.1** | -1.51 | 0.007 |  |
| 12915547 |  | **349.7** | **530.1** | -1.51 | 0.007 |  |
| 12912591 |  | **13.5** | **20.4** | -1.51 | 0.009 |  |
| 12841045 | GBP4 | **9.8** | **14.8** | -1.52 | 0.008 | guanylate binding protein 4 |
| 12760136 | LOC789715 | **17.9** | **27.1** | -1.52 | 0.022 | zinc finger protein 548-like |
| 12911215 |  | **302.3** | **458.3** | -1.52 | 0.011 |  |
| 12915463 |  | **302.3** | **458.3** | -1.52 | 0.011 |  |
| 12885762 | ANGPTL6 | **40.8** | **62.2** | -1.53 | 0.011 | angiopoietin-like 6 |
| 12848299 | ARL4C | **29.9** | **45.6** | -1.53 | 0.021 | ADP-ribosylation factor-like 4C |
| 12767314 | KCNJ2 | **21.0** | **32.0** | -1.53 | 0.049 | potassium inwardly-rectifying channel, subfamily J, member 2 |
| 12809935 | PSTPIP2 | **10.7** | **16.6** | -1.54 | 0.027 | proline-serine-threonine phosphatase interacting protein 2 |
| 12911189 |  | **604.7** | **935.8** | -1.54 | 0.002 |  |
| 12911191 |  | **604.7** | **935.8** | -1.54 | 0.002 |  |
| 12911193 |  | **604.7** | **935.8** | -1.54 | 0.002 |  |
| 12915537 |  | **604.7** | **935.8** | -1.54 | 0.002 |  |
| 12915539 |  | **604.7** | **935.8** | -1.54 | 0.002 |  |
| 12915541 |  | **604.7** | **935.8** | -1.54 | 0.002 |  |
| 12850265 | TMEM140 | **31.8** | **49.2** | -1.55 | 0.020 | transmembrane protein 140 |
| 12770584 |  | **15.6** | **24.1** | -1.55 | 0.015 |  |
| 12791056 | GABRG3 | **4.9** | **7.6** | -1.56 | 0.021 | gamma-aminobutyric acid (GABA) A receptor, gamma 3 |
| 12822975 | HSPA12A | **31.3** | **48.8** | -1.56 | 0.013 | heat shock 70kDa protein 12A |
| 12915515 |  | **101.1** | **157.6** | -1.56 | 0.013 |  |
| 12906699 | NRK | **11.0** | **17.3** | -1.57 | 0.040 | Nik related kinase |
| 12911157 |  | **436.5** | **685.0** | -1.57 | 0.005 |  |
| 12913073 |  | **21.0** | **32.9** | -1.57 | 0.024 |  |
| 12767029 | CCL8 | **17.9** | **28.2** | -1.58 | 0.047 | chemokine (C-C motif) ligand 8 |
| 12884126 | OR1I1 | **7.3** | **11.5** | -1.58 | 0.045 | olfactory receptor, family 1, subfamily I, member 1 |
| 12867949 | PDGFB | **41.9** | **66.3** | -1.58 | 0.002 | platelet-derived growth factor beta polypeptide |
| 12794204 | SEMA3G | **47.2** | **74.5** | -1.58 | 0.034 | sema domain, immunoglob. in domain (Ig), short basic domain, secr., (semaphorin) 3G |
| 12831421 | PAK1 | **50.2** | **79.9** | -1.59 | 0.042 | p21 protein (Cdc42/Rac)-activated kinase 1 |
| 12910359 | RPL39 | **458.3** | **729.1** | -1.59 | 0.005 | ribosomal protein L39 |
| 12912941 |  | **8.0** | **12.7** | -1.59 | 0.004 |  |
| 12721780 | ASAP1 | **61.8** | **98.4** | -1.60 | 0.033 | ArfGAP with SH3 domain, ankyrin repeat and PH domain 1 |
| 12882031 |  | **40.5** | **65.3** | -1.61 | 0.019 |  |
| 12794373 | RPSA | **374.8** | **608.9** | -1.62 | 0.003 | ribosomal protein SA |
| 12848304 |  | **10.9** | **17.6** | -1.62 | 0.001 |  |
| 12775239 | CFLAR | **78.8** | **128.0** | -1.63 | 0.035 | CASP8 and FADD-like apoptosis regulator |
| 12699482 | DUSP11 | **194.0** | **317.4** | -1.64 | 0.040 | dual specificity phosphatase 11 (RNA/RNP complex 1-interacting) |
| 12751471 | CPT1C | **29.4** | **49.2** | -1.66 | 0.016 | carnitine palmitoyltransferase 1C |
| 12802001 | HIST1H1C | **29.9** | **49.9** | -1.67 | 0.047 | histone cluster 1, H1c |
| 12815240 | CORO1A | **75.1** | **126.2** | -1.68 | 0.029 | coronin, actin binding protein, 1A |
| 12913979 |  | **9.8** | **16.9** | -1.72 | 0.013 |  |
| 12914853 |  | **19.2** | **32.9** | -1.72 | 0.044 |  |
| 12849428 |  | **4.6** | **8.0** | -1.73 | 0.045 |  |
| 12836105 | GNG12 | **152.2** | **265.0** | -1.74 | 0.048 | guanine nucleotide binding protein (G protein), gamma 12 |
| 12680936 | MASP1 | **17.9** | **31.1** | -1.74 | 0.007 | mannan-binding lectin serine peptidase 1 (C4/C2 activating component of Ra-reactive factor) |
| 12879457 | MARCH3 | **26.2** | **45.6** | -1.75 | 0.025 | membrane-associated ring finger (C3HC4) 3 |
| 12756042 | PEG3; bta-mir-2900 | **70.0** | **124.5** | -1.78 | 0.017 | paternally expressed 3; bta-mir-2900 |
| 12728090 |  | **56.1** | **100.4** | -1.78 | 0.025 |  |
| 12804076 | JSP.1; BOLA; BOLA-N; LOC100125916 | **284.0** | **512.0** | -1.80 | 0.023 | MHC Class I JSP.1; MHC class I heavy chain; MHC class I antigen; uncharacterized protein 100125016 |
| 12851804 | TMEM243 | **44.3** | **79.9** | -1.80 | 0.037 | chromosome 4 open reading frame, human C7orf23 |
| 12804085 |  | **284.0** | **512.0** | -1.80 | 0.023 |  |
| 12818707 | IFIT1 | **14.5** | **26.4** | -1.82 | 0.024 | interferon-induced protein with tetratricopeptide repeats 1 |
| 12787702 | IL7R | **8.8** | **16.0** | -1.82 | 0.050 | interleukin 7 receptor |
| 12683071 | LSAMP | **13.5** | **24.8** | -1.85 | 0.039 | neuronal growth regulator 1-like |
| 12901108 | PRDM1 | **29.0** | **54.2** | -1.88 | 0.025 | PR domain containing 1, with ZNF domain |
| 12721237 | JAG1 | **106.9** | **202.3** | -1.89 | 0.045 | jagged 1 |
| 12785106 | MYO10 | **41.6** | **81.6** | -1.96 | 0.006 | myosin X |
| 12911107 |  | **280.1** | **548.7** | -1.96 | 0.011 |  |
| 12912457 |  | **3.5** | **6.8** | -1.96 | 0.021 |  |
| 12736063 | HMCN1 | **47.5** | **95.7** | -2.02 | 0.019 | hemicentin 1 |
| 12732807 | MICAL2 | **20.0** | **40.2** | -2.02 | 0.047 | microtubule associated monoxygenase, calponin and LIM domain containing 2 |
| 12678517 | MX1 | **117.0** | **235.6** | -2.02 | 0.003 | myxovirus (influenza virus) resistance 1, interferon-inducible protein p78 (mouse) |
| 12843823 |  | **11.6** | **23.3** | -2.02 | 0.046 |  |
| 12872717 | TIFA | **20.1** | **40.8** | -2.03 | 0.049 | TRAF-interacting protein with forkhead-associated domain |
| 12740864 | RGS1 | **24.6** | **50.6** | -2.05 | 0.030 | regulator of G-protein signaling 1 |
| 12679430 | COL18A1 | **39.9** | **82.1** | -2.06 | 0.030 | collagen, type XVIII, alpha 1 |
| 12748219 | OAS1Y | **76.6** | **158.7** | -2.06 | 0.011 | 2',5'-oligoadenylate synthetase 1, 40/46kDa |
| 12848811 | AHR | **111.4** | **230.7** | -2.07 | 0.022 | aryl hydrocarbon receptor |
| 12905109 | DOCK11 | **26.2** | **56.1** | -2.15 | 0.031 | dedicator of cytokinesis 11 |
| 12871206 | PARM1 | **38.3** | **83.9** | -2.20 | 0.009 | prostate androgen-regulated mucin-like protein 1 |
| 12744361 | PDGFC | **93.1** | **207.9** | -2.23 | 0.010 | platelet derived growth factor C |
| 12776406 | IFI6 | **648.1** | **1458.2** | -2.24 | 0.024 | interferon, alpha-inducible protein 6 |
| 12911675 |  | **3.2** | **7.4** | -2.30 | 0.015 |  |
| 12911705 |  | **3.2** | **7.4** | -2.30 | 0.015 |  |
| 12708928 | ABCC4 | **3.3** | **8.1** | -2.43 | 0.023 | ATP-binding cassette, sub-family C (CFTR/MRP), member 4 |
| 12789530 | IFI27 | **157.6** | **390.7** | -2.48 | 0.048 | putative ISG12(a) protein |
| 12836660 | OLFML3 | **21.9** | **56.9** | -2.61 | 0.042 | olfactomedin-like 3 |
| 12856897 | OLR1 | **14.2** | **39.4** | -2.76 | 0.031 | oxidized low density lipoprotein (lectin-like) receptor 1 |
| 12678626 | ALCAM | **43.4** | **123.6** | -2.86 | 0.008 | activated leukocyte cell adhesion molecule |
| 12730744 | IL18 | **86.2** | **250.7** | -2.89 | 0.041 | interleukin 18 (interferon-gamma-inducing factor) |
| 12731703 | FXYD6 | **240.5** | **719.1** | -2.99 | 0.005 | FXYD domain containing ion transport regulator 6 |
| 12798575 | BHLHE40 | **107.6** | **340.1** | -3.16 | 0.039 | basic helix-loop-helix family, member e40 |
| 12885799 | IFI47 | **14.2** | **110.7** | -7.76 | 0.042 | interferon gamma inducible protein 47 |

Mean hybridization signals (Signal), fold change (FC) and p-value from unpaired one-way ANOVA statistical analysis are shown.
