# Supplementary material for: Exposure of Lactating Dairy Cows to Acute Pre-Ovulatory Heat Stress Affects Granulosa Cell-Specific Gene Expression Profiles in Dominant Follicles
Source: PLoS One. 2016 Aug 17;11(8):e0160600. doi: 10.1371/journal.pone.0160600 (PMC4988698; doi:10.1371/journal.pone.0160600)
Supplement: S4 Table — (DOCX) [file pone.0160600.s004.docx]

S4 Table. Significantly affected Canonical Pathways according to IPA analysis.

| **#** | **Ingenuity Canonical Pathways** | **p-value** | **Ratio** | **Molecules** |
| --- | --- | --- | --- | --- |
| 1 | Sphingosine-1-phosphate Signaling | 0.000 | 0.102 | PIK3C2A, ADCY6, SMPD3, PLCD4, PDGFC, PDGFB |
| 2 | Mitochondrial L-carnitine Shuttle Pathway | 0.001 | 0.231 | SLC27A1, SLC27A3, CPT1C |
| 3 | Glycerol Degradation I | 0.001 | 0.500 | GK, GPD2 |
| 4 | PAK Signaling | 0.002 | 0.089 | ARHGAP10, PIK3C2A, PDGFC, PDGFB |
| 5 | LPS/IL-1 Mediated Inhibition of RXR Function | 0.003 | 0.053 | IL18, NR1H4, CHST11, SLC27A1, SLC27A3, CPT1C |
| 6 | Fatty Acid Activation | 0.005 | 0.222 | SLC27A1, SLC27A3 |
| 7 | P2Y Purigenic Receptor Signaling Pathway | 0.006 | 0.069 | PIK3C2A, ADCY6, PLCD4, GNG12 |
| 8 | Macropinocytosis Signaling | 0.008 | 0.094 | PIK3C2A, PDGFC, PDGFB |
| 9 | Antiproliferative Role of Somatostatin Receptor 2 | 0.008 | 0.091 | PIK3C2A, NPR1, GNG12 |
| 10 | Actin Cytoskeleton Signaling | 0.009 | 0.050 | PIK3C2A, WASF1, PDGFC, PDGFB, GNG12 |
| 11 | γ-linolenate Biosynthesis II (Animals) | 0.010 | 0.167 | SLC27A1, SLC27A3 |
| 12 | PPARα/RXRα Activation | 0.010 | 0.048 | GK, GPD2, ADCY6, SLC27A1, PLCD4 |
| 13 | Relaxin Signaling | 0.012 | 0.057 | PIK3C2A, NPR1, ADCY6, GNG12 |
| 14 | GPCR-Mediated Nutrient Sensing in Enteroendocrine Cells | 0.012 | 0.079 | ADCY6, PLCD4, GNG12 |
| 15 | Type II Diabetes Mellitus Signaling | 0.013 | 0.056 | PIK3C2A, SLC27A1, SLC27A3, SMPD3 |
| 16 | Glioblastoma Multiforme Signaling | 0.016 | 0.053 | PIK3C2A, PLCD4, PDGFC, PDGFB |
| 17 | Gap Junction Signaling | 0.016 | 0.053 | PIK3C2A, NPR1, ADCY6, PLCD4 |
| 18 | Leptin Signaling in Obesity | 0.016 | 0.071 | PIK3C2A, ADCY6, PLCD4 |
| 19 | PDGF Signaling | 0.019 | 0.067 | PIK3C2A, PDGFC, PDGFB |
| 20 | CREB Signaling in Neurons | 0.019 | 0.049 | PIK3C2A, ADCY6, PLCD4, GNG12 |
| 21 | UVA-Induced MAPK Signaling | 0.020 | 0.065 | PIK3C2A, SMPD3, PLCD4 |
| 22 | Circadian Rhythm Signaling | 0.021 | 0.111 | ARNTL, BHLHE40 |
| 23 | Atherosclerosis Signaling | 0.023 | 0.063 | IL18, PDGFC, PDGFB |
| 24 | Fatty Acid β-oxidation I | 0.024 | 0.105 | SLC27A1, SLC27A3 |
| 25 | Glycerol-3-phosphate Shuttle | 0.025 | 0.500 | GPD2 |
| 26 | Stearate Biosynthesis I (Animals) | 0.032 | 0.091 | SLC27A1, SLC27A3 |
| 27 | Superpathway of Inositol Phosphate Compounds | 0.032 | 0.043 | PIK3C2A, STYXL1, PLCD4, PXYLP1 |
| 28 | Corticotropin Releasing Hormone Signaling | 0.032 | 0.055 | NPR1, PTCH1, ADCY6 |
| 29 | Role of Macrophages, Fibroblasts and Endothelial Cells in Rheumatoid Arthritis | 0.032 | 0.036 | IL18, PIK3C2A, PLCD4, PDGFC, PDGFB |
| 30 | Glioma Signaling | 0.034 | 0.054 | PIK3C2A, PDGFC, PDGFB |
| 31 | Thrombin Signaling | 0.036 | 0.041 | PIK3C2A, ADCY6, PLCD4, GNG12 |
| 32 | Role of NFAT in Cardiac Hypertrophy | 0.036 | 0.041 | PIK3C2A, ADCY6, PLCD4, GNG12 |
| 33 | Tetrapyrrole Biosynthesis II | 0.038 | 0.333 | ALAD |
| 34 | NAD Phosphorylation and Dephosphorylation | 0.038 | 0.333 | PXYLP1 |
| 35 | PPAR Signaling | 0.043 | 0.049 | IL18, PDGFC, PDGFB |
| 36 | GABA Receptor Signaling | 0.043 | 0.077 | GABRG3, ADCY6 |
| 37 | Axonal Guidance Signaling | 0.047 | 0.029 | PIK3C2A, PTCH1, PLCD4, PDGFC, PDGFB, GNG12 |
| 38 | Human Embryonic Stem Cell Pluripotency | 0.048 | 0.047 | PIK3C2A, PDGFC, PDGFB |
